# Toward Omics-Scale Quantitative Mass Spectrometry Imaging of Lipids in Brain Tissue Using a Multiclass Internal Standard Mixture

**DOI:** 10.1021/acs.analchem.3c02724

**Published:** 2023-12-11

**Authors:** Michiel Vandenbosch, Shadrack M. Mutuku, Maria José Q. Mantas, Nathan H. Patterson, Tucker Hallmark, Marc Claesen, Ron M. A. Heeren, Nathan G. Hatcher, Nico Verbeeck, Kim Ekroos, Shane R. Ellis

**Affiliations:** † Maastricht MultiModal Molecular Imaging (M4I) Institute, Division of Imaging Mass Spectrometry, Maastricht University, Maastricht 6229ER, Netherlands; ‡ Molecular Horizons and School of Chemistry and Molecular Bioscience, University of Wollongong, Wollongong, NSW 2522, Australia; § Aspect Analytics NV, Genk 3600, Belgium; ∥ Avanti Polar Lipids, Alabama, Alabama 35007, United States; ⊥ Merck & Co., Inc., 770 Sumneytown Pk, West Point, Pennsylvania 19486, United States; # Lipidomics Consulting Ltd., Esbo 02230, Finland

## Abstract

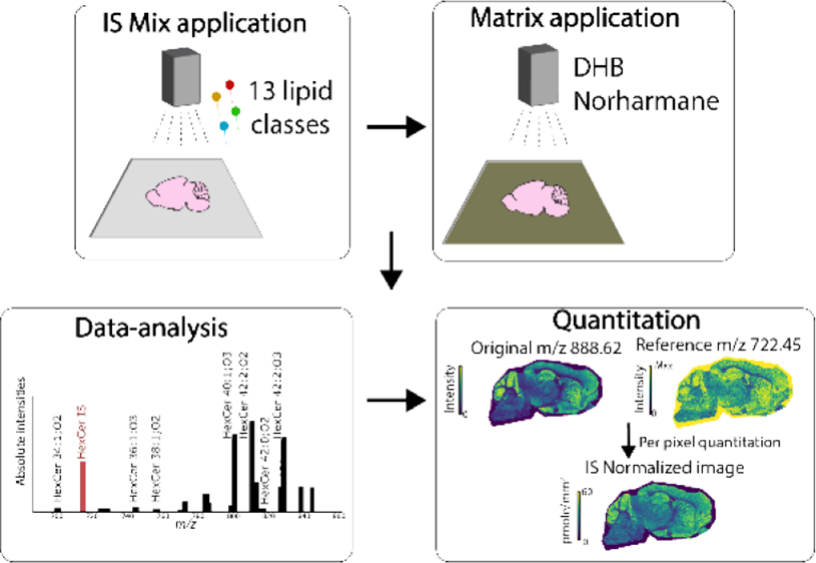

Mass spectrometry imaging (MSI) has accelerated our understanding of lipid metabolism and spatial distribution in tissues and cells. However, few MSI studies have approached lipid imaging quantitatively and those that have focused on a single lipid class. We overcome this limitation by using a multiclass internal standard (IS) mixture sprayed homogeneously over the tissue surface with concentrations that reflect those of endogenous lipids. This enabled quantitative MSI (Q-MSI) of 13 lipid classes and subclasses representing almost 200 sum-composition lipid species using both MALDI (negative ion mode) and MALDI-2 (positive ion mode) and pixel-wise normalization of each lipid species in a manner analogous to that widely used in shotgun lipidomics. The Q-MSI approach covered 3 orders of magnitude in dynamic range (lipid concentrations reported in pmol/mm^2^) and revealed subtle changes in distribution compared to data without normalization. The robustness of the method was evaluated by repeating experiments in two laboratories using both timsTOF and Orbitrap mass spectrometers with an ∼4-fold difference in mass resolution power. There was a strong overall correlation in the Q-MSI results obtained by using the two approaches. Outliers were mostly rationalized by isobaric interferences or the higher sensitivity of one instrument for a particular lipid species. These data provide insight into how the mass resolving power can affect Q-MSI data. This approach opens up the possibility of performing large-scale Q-MSI studies across numerous lipid classes and subclasses and revealing how absolute lipid concentrations vary throughout and between biological tissues.

## Introduction

Mass spectrometry imaging (MSI) is a powerful tool for mapping the spatial distribution of lipids^1,2^ and other analytes throughout biological tissues.^3−5^ Lipids are one of the most common target analytes for MSI, in part due to the relative ease by which several lipid classes are detected but primarly given the vital role of lipid metabolism in many biological functions and diseases.^6−8^ MSI of lipids has thus been applied in diverse biological applications, including studying solid cancers,^9−11^ neurodegenerative disorders,^12,13^ cardiovascular disease,^14,15^ fatty liver disease,^16^ and lung disease.^17^ Furthermore, recent developments in postionization strategies coupled–matrix-assisted laser desorption/ionization (MALDI) such as laser-postionization (MALDI-2),^18^ plasma postionization,^19−21^ and vacuum-ultraviolet-based methods^22^ have significantly expanded the number of lipid classes that can be studied with MSI.^23^

MSI data visualization is based on the mapping of ion intensities across the samples, often following normalization procedures based on either total-ion current (TIC) or root-mean-square (RMS) normalization.^24^ However, the ion intensities for different analytes are not necessarily proportional to their concentrations. As a result, two analytes present at identical concentrations may have differing signal intensities. These effects can vary significantly depending on the desorption/ionization method employed, the nature of the analytes, and the sample environment from which they are analyzed and are not corrected for through conventional TIC or RMS normalization. In the case of lipids, ionization efficiencies are strongly dependent on the lipid class and the presence of other lipid classes, meaning ion intensities vary by several orders of magnitude across different classes. Regarding MSI, the variation in the chemical and morphological environment present at each sampling position determines the extent of matrix effects.^25−27^ This can also lead to discrepancies in ion intensities for a given lipid species across a tissue, even if present at equal concentrations.^28,29^ Thus, while MSI has shown much versatility for elucidating region-specific lipid fingerprints and relative changes across tissues, it does not typically allow for absolute quantitation (i.e., the analyte concentration per unit area or volume). Similar challenges in obtaining quantitative data also arise in both shotgun and LC-MS/MS-based lipidomics studies. Currently, these issues are addressed by using class-specific stable isotope labeled (SIL) or non-endogenous internal standards (IS).^30−32^ These are introduced prior to sample homogenization and extractions at a single concentration to account for potential lipid losses during sample preparation/analyte recovery and class-specific ionization behaviors by normalizing endogenous lipid signals to their respective class-specific IS. For a given lipid class, the IS should have an identical or near-identical ionization efficiency as endogenous lipids of the same class.^33^ Thus, the ratio of endogenous lipid signal to the IS allows for absolute or accurate quantitation of the given lipid class when the IS concentration is known.^34^ The majority of lipidomics studies utilize a single internal concentration for each class and this approach has been widely adopted,^32,35^ including for shotgun and LC-MS approaches.^36,37^ Moreover, single point calibration methods have been shown to agree well to quantitative data generated using class-specific calibration lines.^38^ MSI can be considered as an analog of shotgun lipidomics in that the sample is analyzed in the absence of any chromatography; thus, approaches deployed for shotgun lipidomics should be applicable to MSI.

Similar approaches have been adapted for quantitative MSI (Q-MSI).^39^ In such approaches, a suitable IS (again, typically a SIL analog of the compound of interest) is sprayed evenly across a tissue sample, and the endogenous signal is normalized to the IS signal in each pixel. Q-MSI has been most widely used in the pharmacological context for drug imaging, including imatinib,^40^ clozapine,^41^ and rifampicin,^42^ among others.^43^ In pharmacokinetic/pharmacodynamics studies, knowing the spatial distribution of pharmaceutical compounds and their localized concentrations is key to a deeper understanding of drug-target engagement, metabolism, biomarker response, changes in the tumor microenvironment, and/or resolution of tissue injury.^39^ However, it is not possible to assess variations in matrix effects across different regions of the analyzed tissue using homogenates. Crucially, Q-MSI has been shown to correlate well with LC-MS/MS data, e.g., the antipancreatic cancer drug gemcitabine^44^ and drug candidates in dog liver^45^ using desorption electrospray ionization (DESI)-MSI and donepezil hydrochloride^46^ in mice brain by MALDI-MSI. Quantitative amounts obtained by Q-MSI often lie within 5%–20% of those obtained using regional dissections^47^ and LC-MS,^41^ corroborating the validity of the approach.

Q-MSI has been applied using nano-DESI to measure the abundance of phosphatidylcholine (PC) species in specific regions of rat brain tissue sections.^48^ The extraction efficiency of the spray, which depends on the structural properties of the tissue, was critical in the estimation of absolute amounts of PC species throughout brain tissue and was corrected for using internal standards.^48^ In another example of Q-MSI of lipids, cholesterol was quantified in brain tissue in a mouse model of Niemann-Pick type 1 disease.^49^ A limitation of Q-MSI studies to date has been the targeted nature of the analysis, meaning only several targeted analytes or a targeted analyte class (e.g., PC lipids) could be studied, due to the use of a single IS. As MSI is akin to performing a shotgun lipidomics analysis at each pixel, here we have adapted methods from conventional shotgun lipidomics^34^ for quantitation of multiple lipid classes and subclasses. We have developed an IS mixture for MSI of brain tissue that contains either SIL, or non-endogenous odd-chain species of 13 lipid classes and subclasses of lipids belonging to the glycerophospholipid and sphingolipid lipid categories that are ubiquitous in brain tissue. Using a combination of MALDI and MALDI-2 as well as orthogonal-time-of-flight and Orbitrap mass analyzers, we demonstrate the ability to perform Q-MSI on almost 200 lipid species across positive- and negative-ion mode analyses and investigate the influence of mass resolution on the accuracy of quantitative results.

## Methods

### Chemicals

At the University of Wollongong (UOW), LC-MS hypergrade methanol, chloroform, norharmane, and 2,5-dihydroxybenzoic acid (DHB) were purchased from Merck/Sigma-Aldrich (North Ryde NSW, Australia). Haematoxylin and eosin, 1% aqueous, were purchased from Point of Care Diagnostics (NSW, Australia). Xylene and quick-hardening mounting medium for microscopy were purchased from Sigma-Aldrich (NSW, Australia).

At Maastricht University (UM), water, methanol, and chloroform (all HPLC and ULC/MS grade) were obtained from Biosolve B.V. (Valkenswaard, The Netherlands). Norharmane and DHB were obtained from Sigma-Aldrich (St. Louis, MI, USA). Hematoxylin and Entellan were obtained from Merck (Darmstadt, Germany). Eosin Y was obtained from J.T. Baker (Center Valley, PA, USA).

The internal standard mixture (product number #330841 MSI SPLASH) consists of standards for 13 lipid classes and subclasses derived from glycerophospholipid and sphingolipid lipid categories developed in collaboration with AVANTI Polar Lipids (Alabaster, Alabama, USA). The composition of the IS mixture used for this work is listed in Table 1.

**Table 1:** Lipid Composition of the Internal Standard Mixture Used^a^

**mixture component**	**concentration**(mg/mL)	**adduct**	*m*/*z*
15:0–18:1 (d7) PA	0.111	[M-H]^−^	666.5097
15:0–18:1 (d7) PE	0.100	[M-H]^−^	709.5519
15:0–18:1 (d7) PG	0.049	[M-H]^−^	740.5465
15:0–18:1 (d7) PI	0.023	[M-H]^−^	828.5625
17:0–16:1 PS-d5	0.105	[M-H]^−^	751.5291
17:0 Lyso PE-d5	0.003	[M-H]^−^	471.3253
C12 Mono-Sulfo Galactosyl(ß) Ceramide (d18:1/12:0)	0.019	[M-H]^−^	722.4519
C15 Lactosyl(β) Ceramide-d7 (d18:1-d7/15:0)	0.013	[M+H]^+^	855.6533
C18 Ceramide-d7 (d18:1/18:0)	0.011	[M+H]^+^	573.5946
C17 Glucosyl(ß) Ceramide (d18:1/17:0)	0.133	[M+H]^+^	714.5878
d18:1–18:1 (d9) SM	0.031	[M+H]^+^	738.6470
17:0 Lyso PC-d5	0.003	[M+H]^+^	515.3868
15:0–18:1 (d7) PC	0.161	[M+H]^+^	753.6134
	0.161	[M+Na]^+^	775.5953

a Note the more abundant [M+K]^+^ adduct for the PC IS was not used due to overlap with an endogenous lipid signal produced by MALDI-2.

### Sample Preparation

Fresh frozen brain specimens from wild-type (WT) C57BL/6N eight-month aged mice (Jackson Laboratory, ME, USA) were provided by Merck & Co., Inc., Rahway, NJ, USA. Animals were singly housed, had access to food and water *ad libitum*, and were kept on a 12/12 h light/dark cycle in a temperature (22 ± 2 °C) and humidity (∼50%) controlled facility. Mice were anaesthetized with 3% isoflurane prior to euthanasia, and intact brain specimens were isolated and immediately frozen under dry ice (−80 °C) for shipment for analyses at either UOW or UM. At UOW, frozen brain specimens were sectioned at 12-μm (μm) thickness in a cryostat (CM1950 Leica Biosystems, Germany) on precleaned indium tin oxide (ITO)-coated conductive glass slides (Delta Technologies, CO, USA). Slides were kept frozen in a −80 °C freezer until analyzed. Upon removal, slides were quickly transferred to hygroscopic desiccant (beads)-filled boxes and further vacuum-dried in a desiccation chamber for 20 min.

At UM, tissue sectioning was performed on a Leica CM1860 UV (Wetzlar, Germany) at −20 °C. 12 μm-thick sections were transferred to conductive ITO-coated microscope glass slides (Delta Technologies, Loveland, MN, USA). Slides were stored in a −80 °C freezer until analyzed and vacuum-dried as described above. All animal studies were performed under the approval of the Merck & Co., Inc., Rahway, NJ, USA, Institutional Animal Care and Use Committee and endorsed by the Animal Ethics Committee of UOW. For the research conducted at UM, an exemption was granted for an ethics application and the study received approval.

### Internal Standard and Matrix Application

At both institutes, the internal standard mixture (IS mix) was diluted 10-fold in LC-MS grade methanol prior to deposition onto tissue sections. An off-line 2.5 mL Leur lock gas-tight syringe (Trajan Scientific, Victoria, Australia) in combination with a syringe pump was connected to the TM-Sprayer (HTX Technologies, USA) for spray coating of the MALDI SPLASH mix. The settings for IS mix deposition were as follows: temperature, 50 °C; number of passes, 16 layers; flow rate, 60 μL/min; velocity, 1200 mm/min; track spacing, 2 mm; gas flow rate 10 psi, 2 L/min and drying time in between passes, 30 s. Identical spraying parameters were used across both laboratories at UOW and UM. The amount of IS deposited in μg/mm^2^ was calculated by multiplying the analyte concentration (μg/mL), flow rate (mL/min), time (min), surface area sprayed (mm^2^), and number of passes (layers). This value was then divided by the average molecular weight (Da) and the dilution factor, yielding the concentration per unit area expressed as picomoles per mm^2^ (pmol/mm^2^). The resulting concentrations for each IS are provided in Supporting Information Table S1. Slides were then immediately coated with the MALDI matrix. Norharmane and DHB matrices were used for negative and positive ion mode imaging, respectively, and were both dissolved in a chloroform–methanol mixture (2:1 v/v). The matrix was deposited on tissue samples using the same TM-Sprayer (HTX Technologies, USA). For negative ion mode analyses, the spray settings were: matrix concentration, 7 mg/mL norharmane; temperature, 30 °C; number of passes, 15 layers; flow rate, 120 μL/min; velocity, 1200 mm/min; track spacing, 3 mm; gas flow rate, 10 psi; and time in between passes, 30 s. For positive ion mode analyses, the spray parameters were: matrix concentration, 15 mg/mL DHB; temperature, 30 °C; number of passes, 10 layers; flow rate, 120 μL/min; velocity, 1200 mm/min; track spacing, 3 mm; gas flow rate, 2 L/min, and time in between passes, 30 s.

### Lipid Nomenclature

Lipids are named according to the most recent LIPIDMAPS guidelines.^50^ We refer throughout to both lipid classes and subclasses as different lipid families that have been analyzed by Q-MSI. Some belong to either their own class (e.g., Cer, HexCer, Hex2Cer and SM), while others are subclasses of different phospholipid classes (e.g., PE, PE-O, and LPE are subclasses of the PE class).

### Mass Spectrometry Imaging

#### Orbitrap Elite

At UOW, tissue sections were analyzed using an Orbitrap Elite mass spectrometer (Thermo Fisher Scientific GmbH, Bremen, Germany) equipped with a dual MALDI/ESI Injector (Spectroglyph LLC, Kennewick, WA, USA). All acquisitions were conducted at a pixel size of 75 μm (*x*, *y*), MALDI laser pulse energy of 1.0 μJ/pulse (measured after an external attenuator), an injection time of 250 ms, automatic gain control turned off, and mass resolution of 240,000 @ *m*/*z* 400. Negative ion mode analysis was conducted using conventional MALDI (i.e., without MALDI-2) using a laser repetition rate of 500 Hz and a mass range of *m*/*z* 180–2,000. Positive-ion mode analysis was conducted using MALDI-2 and a mass range of *m*/*z* 350–2,000. Laser postionization was achieved using a 266 nm laser (NanoDPSS, Litron lasers, Rugby, UK) operating with 500 μJ pulses entering the ion source. Both lasers were operated at 300 Hz and an interpulse delay of 20 μs. Further details on the MALDI-2 setup can be found elsewhere.^51^

#### MALDI-2-timsTOF Flex

At UM, tissue sections were imaged on a MALDI-2 timsTOF flex (Bruker Daltonik, Bremen, Germany). The mass resolution of this instrument is calculated to be 54,000 at *m*/*z* 700. Primary ionization of the material was achieved with a SmartBeam 3D 355 nm laser. Data were acquired at a pixel size of 30 μm (*x*, *y*) using a beam scan area of 26 × 26 μm and a mass range of *m*/*z* 300–1000. For negative mode measurements, the laser was operated at 10 kHz with 200 shots accumulated per pixel and data acquired across a mass range of *m*/*z* 300–1000. For positive mode measurements using MALDI-2, both lasers were operated at 1 kHz with 50 laser shots accumulated per pixel and data acquired across a mass range of *m*/*z* 350–1,000. The MALDI-2 laser (NL204–1K-FH, Ekspla, Lithuania) was operated at 500 μJ/pulse with a pulse delay time of 10 μs. The instrument was calibrated using red phosphorus prior to each measurement. In both cases, glass slides after MSI were subjected to hematoxylin and eosin (H&E) staining method (see protocol in the Supporting Information). For both Orbitrap and timsTOF Flex data was acquired from three biological replicates sent to each lab (i.e., UOW and UM; 6 mouse brain samples in total).

### Data Processing and Analyses

#### Orbitrap Elite Lipid Identification Target Lists

Orbitrap raw data was internally recalibrated using Recal Offline software. Masses used for recalibration are provided in Supporting Information Table S2. MSI files were then converted to mzML using Proteowizard msConvert GUI^52^ before conversion to imzML using LipostarMSI software (Molecular Horizon Srl, Perugia, Italy).^53^ Data were imported into LipostarMSI to generate initial lipid target lists. Import parameters were as follows: Savitzky–Golay smoothing was performed at window size, 7 points; degree, 2; iterations, 1; for peak picking the minimum SNR was set at 0.00; noise window size, 0.10 amu; minimum absolute intensity at 0.00. Peaks below 0.50% and 0.20% of the base peak were discarded for negative mode and positive mode, respectively. The *m*/*z* tolerance was set at ±5.00 ppm; minimum peak frequency, 2.00%; spatial chaos, 0.7; isotopic clustering abundance deviation, 30%; and *m*/*z* image correlation threshold, 0.50. Using the resulting peak list, initial lipid identifications were performed using the identification functions in LipostarMSI. A single peak list derived from a merged data set was used for the identification tool and tentative MS1 level lipid annotation are based on the LMSD “bulk” structures database.^50,54^ Identified peaks were filtered to a 3 ppm tolerance, compounds were removed and “approved” as even chain. The resulting ID compounds were further filtered according to lipid subclass and exported as .csv files. The matched ID compounds were manually checked for isobaric lipid sum composition, and peak lists were separated according to adduct types. Identification lists for each lipid class were manually curated and then cross-checked against a recently published in-depth brain lipidomics study.^55^ Lipid species that were not detected in both studies were removed. The full list of lipids is provided in the Supporting Information.

#### timsTOF Data Processing

timsTOF data were internally recalibrated using DataAnalysis software (Bruker, Bremen, Germany). A linear lock mass recalibration was applied using the masses as provided in Supporting Information Table S2. Subsequently, MALDI-MSI data were imported and analyzed in SCiLS lab software (Bruker, Bremen, Germany) where MSI files were converted to imzML for further analysis.

#### Data Analysis and Visualization

Intensities of the various species of interest, i.e., the target lipid species and internal standards, were extracted through binning of the spectra around the computed corresponding *m*/*z* value. For this, a window of 12 and 3 ppm was set for the timsTOF and Orbitrap data, respectively. The maximum intensity per pixel within these windows was taken as the ion intensity for the corresponding pixel. Images were normalized by conducting a pixel-wise division of the original *m*/*z* image by the reference *m*/*z* image (internal standard). The resultant scaled pixel values were multiplied by the corresponding IS concentration to produce an absolute concentration image for each lipid subclass. Pixels with missing values, resulting from zero-division, were replaced by the median value of a surrounding 3 × 3 pixel window. Winsorizing was employed to eliminate hotspots in the images, with the 0.99, 0.75, and 0.99 quantiles for the original *m*/*z* image, reference *m*/*z* image, and IS normalized image, respectively. Winsorizing is a robust method for outlier removal where pixel intensities above a specified quantile are replaced with the value of that quantile.^56^ The reference *m*/*z* images used a different quantile due to the pronounced intensity of the internal standard in the matrix surrounding the tissue. For each MSI experiment, a digital 10×/20× H&E stained whole slide microscopy image of a post-MALDI imaging tissue section was acquired. The web-based digital pathology tool Annotation Studio (Aspect Analytics NV, Genk, Belgium) was used to annotate brain regions in the images with one of five labels: prefrontal cortex/isocortex, midbrain, hindbrain, basal ganglia, and cerebellum.

The MSI and neighboring microscopy images were coregistered using a proprietary landmark-based, nonrigid registration pipeline (Aspect Analytics NV, Genk, Belgium). After registration, the identified anatomical regions of interest (ROIs) were mapped onto the MSI data, facilitating the extraction of ion intensities from the pixels within these ROIs. This enabled the calculation of the mean concentrations for each anatomical region across tissue sections.

Correlation analyses were performed using GraphPad version 10.0. Two-tailed Pearson correlation, computing *r* between the two data sets was applied. Nonlinear regression was applied using least-squares regression and no weighting. The resulting best-fit regression lines with 95% confidence intervals were plotted.

## Results and Discussion

### Optimization of IS Mix Concentration

**Figure 1:**
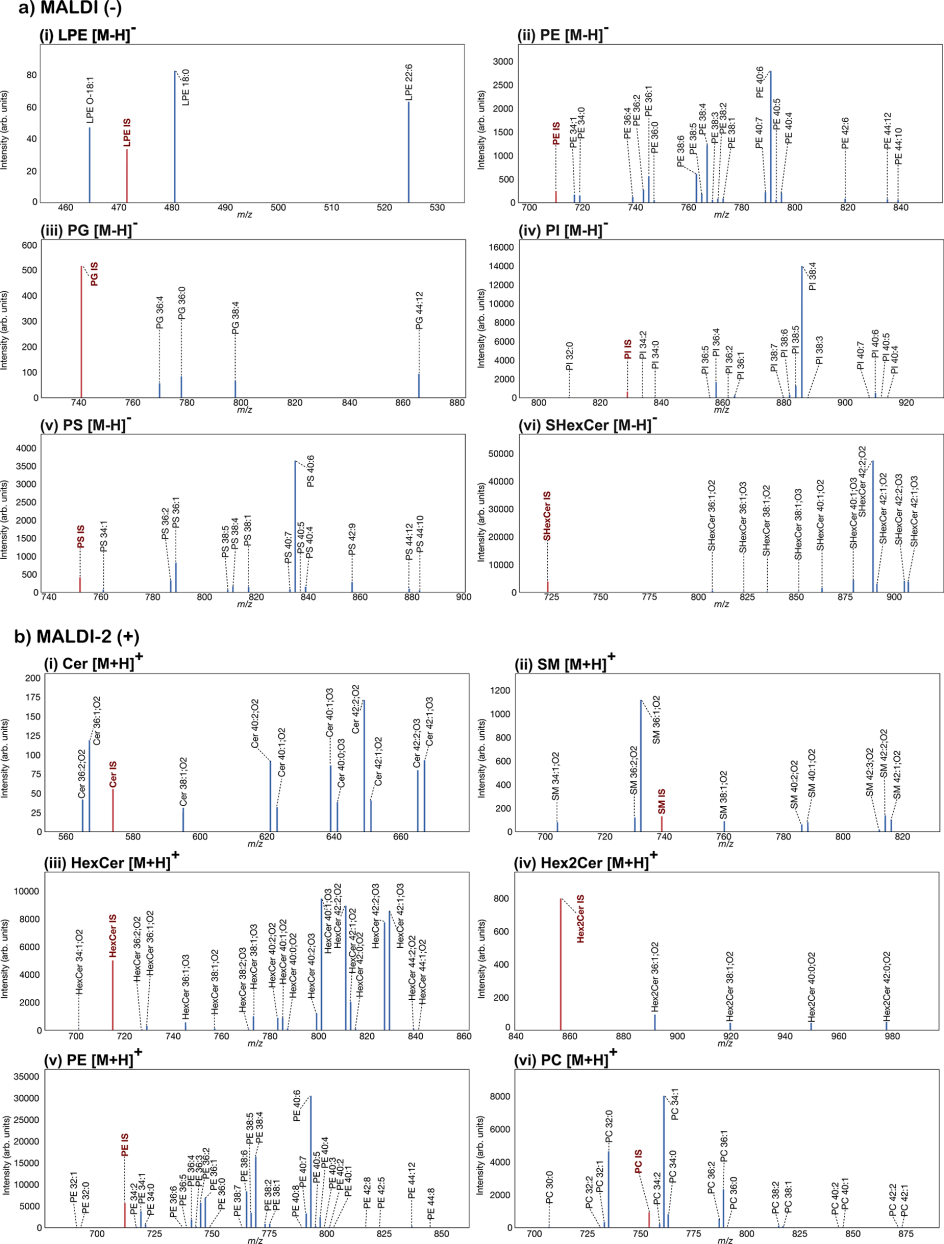
Extracted Orbitrap mass spectra averaged over the entire mouse brain tissue region. (a) Peaks corresponding to (i) LPE, (ii) PE, (iii) PG, (iv) PI, (v) PS, and (vi) SHexCer lipid species detected as [M-H]^−^ ions in negative ion mode. (b) Peaks corresponding to (i) Cer, (ii) SM, (iii) HexCer, (iv) Hex2Cer, (v) PE, and (vi) PC lipid species detected as [M+H]^+^ ions in positive ion mode. Reference IS peak is shown in red, and endogenous lipid species are shown in blue. Similar data for other lipid classes and those acquired using the timsTOF Flex is provided in Supporting Information Figure S2.

A key requirement for the IS mix is that the signal for each lipid standard is reflective of the signals obtained for endogenous lipids of the same subclass. As the abundance of different lipid sublasses varies substantially due to both differing physiological and biochemical concentrations and class-specific ionization efficiencies, the concentration of each IS component must be fine-tuned. The final composition and concentration of the IS mix, shown in Table 1, was determined following an iterative process using both the timsTOF Flex and Orbitrap Elite, which tested for possible interferences with the expected IS peaks. Figure 1a shows extracted negative-ion mode mass spectra of endogenous lipid species for LPE, PE, PG, PI, PS, and SHexCer species with their respective reference internal standard (marked in red) after averaging all on-tissue pixels from one brain section measured using the Orbitrap Elite. For comparative data acquired with the timsTOF Flex and for other lipid classes/subclasses see Supporting Information Figures S1 and S2. As intended, the IS reference peaks for LPE (*m*/*z* 471.3253), PE (*m*/*z* 709.5519), PI (*m*/*z* 828.5625), PS (*m*/*z* 751.5291), and SHexCer (*m*/*z* 722.4519) have abundances similar to moderately abundant endogenous lipid species of each class. Given the low signals of PG species and in order to ensure the detectability of the IS signal in every pixel, the concentration of the PG IS (*m*/*z* 740.5464) was intentionally designed to surpass the most intense endogenous peak.

Figure 1b shows the corresponding positive-ion mode data acquired using MALDI-2 with IS reference observed for the [M+H]^+^ ions of Cer (*m*/*z* 573.5946), SM (*m*/*z* 738.6470), HexCer (*m*/*z* 714.5878), Hex2Cer (*m*/*z* 855.6533), PE (*m*/*z* 711.5664), and PC (*m*/*z* 753.6134). Similar to the PG IS, the higher abundance of the IS for Hex2Cer was deliberately chosen to ensure consistent detection of the IS across the tissue. Unprocessed averaged on-tissue mass spectra in positive and negative ion mode are provided in Supporting Information Figure S3, and a histogram showing the ratios of lipid species to their corresponding IS within the average on-tissue data is provided in Supporting Information Figure S4. The majority of lipid species have intensities within a ±1 order of magnitude of their IS. These data demonstrate the suitability of the chosen concentrations within the IS mix for lipid imaging of brain tissue.

Furthermore, the impact of the internal standard deposition technique on the signals generated for endogenous lipid species was examined through a comparison of three consecutive sections that were subjected to different sprays: no spray, methanol (the solvent for internal standards), and IS deposition. Representative ion images and averaged spectra are shown in Supporting Information Figures S5 and S6. Expectedly, no influence of the IS deposition method on the signals generated for endogenous species was observed.

### Application of Multiclass IS Mix to Lipid MSI

**Figure 2:**
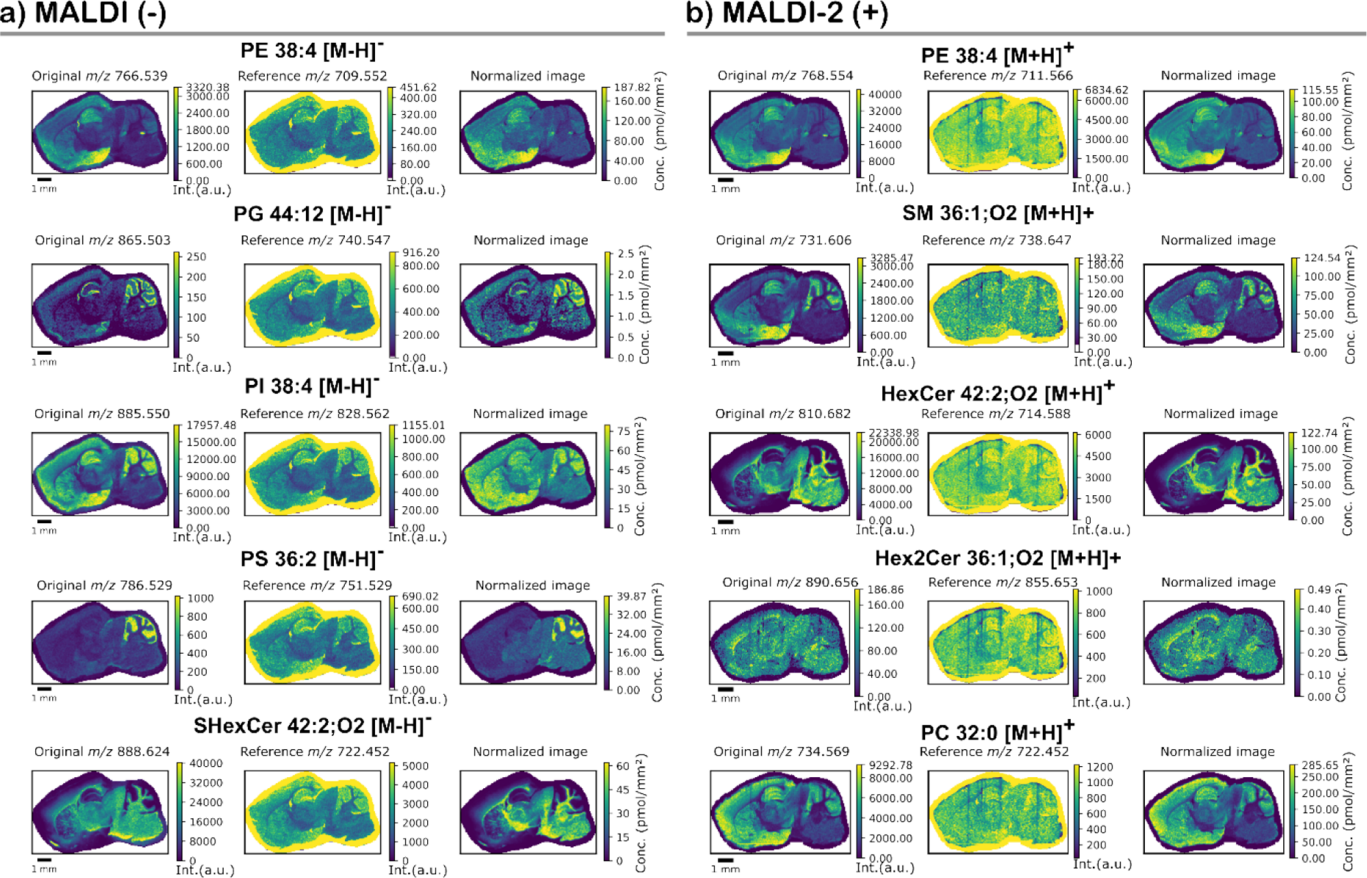
Representative internal standard (IS) normalized ion images for different lipid species detected in (a) negative ion mode using MALDI and (b) positive ion mode using MALDI-2 acquired using the Orbitrap Elite. For each lipid species the original ion image is shown on the left, the class-specific internal standard ion image shown in the center and the IS normalized ion image shown on the right. Intensities for each lipid species were selected using an *m*/*z* window of ±3.0 ppm compared to the theoretical *m*/*z* of the lipid species. (a) Representative negative-ion mode data using MALDI-MSI for the [M-H]^−^ ions of PE 38:4, PG 44:12, PI 38:4, PS 36:2, and SHexCer 42:2;O2. (b) Representative positive ion mode data using MALDI-2-MSI for the [M+H]^+^ ions of PE 38:4, SM 36:1;O2, HexCer 42:2;O2, Hex2Cer 42:2;O2, and PC 36:0. Similar imaging data from the timsTOF Flex can be found in Figure S10.

Next, we evaluated the effect of internal standard (IS) normalization on lipid imaging. The *m*/*z* images were plotted with a ± 3 ppm theoretical mass window of the chosen lipid species for negative ion (Figure 2a) and positive-ion mode (Figure 2b) for the Orbitrap Elite data. For each representative lipid species, the original ion image is shown on the left panel, the IS distributions in the center, and the IS normalized lipid distribution is shown on the right. The reference images show mostly an intense uniform IS distribution in the off-tissue (boundary margin) areas, reflective of the enhanced ionization efficiency of standards and reduced influence of ion suppression when ionized from the glass slide compared to tissue samples. A lower intensity heterogeneous spatial distribution of the internal standards is observed across the on-tissue areas, reflective of both the increased influence of ionization suppression when analyzing the tissue and the region-specific ionization efficiencies that the IS intends to correct. In both negative and positive-ion mode data, the original images are broadly consistent with the IS normalized images; however, subtle differences can be observed that highlight the effect of IS normalization. For example, in negative ion mode analyses, the IS normalized images of PI 38:4 and PS 36:2 ([M-H]^−^ ions) show higher lipid abundance in the brain stem regions compared to the original data (Supporting Information Figure S7). Another benefit of IS normalization is the correction for region-specific distribution of endogenous species to reveal regions of higher concentration. For example, SHexCer 42:2;O2 showed patterns of localization in the white matter (WM) fiber tracts adjacent to the caudoputamen and pallidum inclusive of the cerebellum after IS normalization. Indeed, this correction is beneficial for many lipids including low abundant species such as SHexCer 36:1;O2, which displays a higher contrast distributed within WM regions (Supporting Information Figure S8). This spatial distribution is in line with the literature and the known high abundance of sulfatides and other sphingolipids within myelin.^57^ Additionally, despite the fact that PI 36:2 seems diffusely distributed across the brain without normalization, it appears more precisely localized and pronounced within the WM area of the basal ganglia and brain stem, whereas PI 38:6, initially more intense across the cerebellum, was higher in the hindbrain after IS normalization (Supporting Information Figure S8).

Similar results were obtained in positive ion mode with the distributions of selected PC, SM, PE, HexCer and Hex2Cer shown in Figure 2b. Analogous to Figure 2a, the original (left-hand panels) and IS normalized (right-hand panels) images show the same general distributions with the added benefit of being able to quantify lipid concentrations (see below). Another notable benefit of IS normalization is the ability to correct for streaks in ion images that are sometimes observed using MALDI-2 (Figure 2b). This can be caused by partial blockage of the MALDI-2 laser beam by e.g. a large matrix crystal, slight laser alignment drift or topographical changes on tissue edges. This effect is particularly visible in the images of the [M+H]^+^ ions of the PE, HexCer and Hex2Cer ISs as vertical lines (i.e., the direction of stage raster) in Figure 2. Since the same effect is seen on endogenous species as on the IS, this is corrected after IS normalization. In all, reproducible results were obtained across biological replicates, with several examples shown in Supporting Information Figure S9. Comparative data acquired with the timsTOF Flex are provided in Supporting Information Figure S10.

### Region-Specific Quantitation of Lipids Using Q-MSI

**Figure 3:**
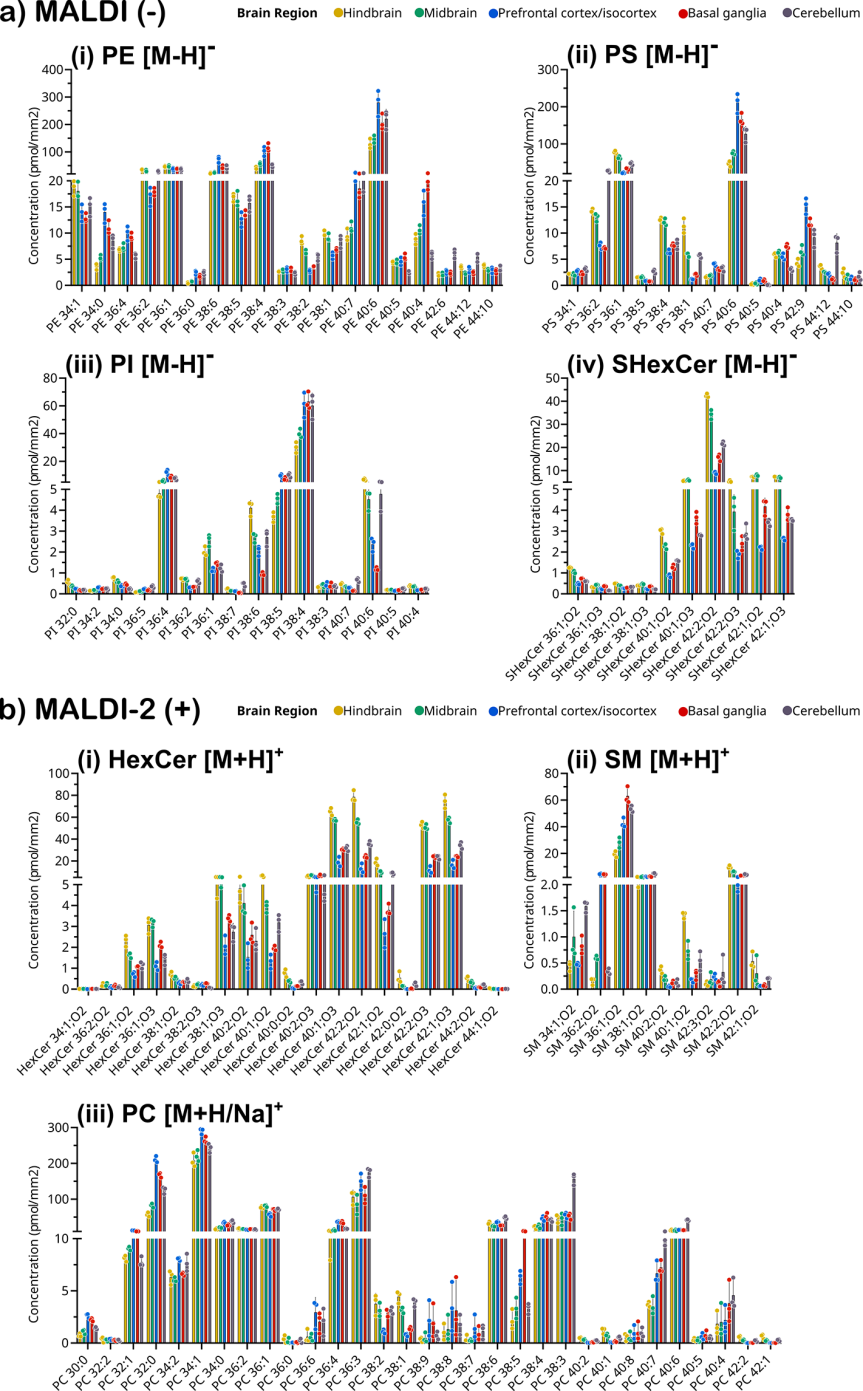
Mean concentration of lipid species from quantitative mass spectrometry imaging in five different brain regions acquired using an Orbitrap Elite. Regions of interest (ROI) from sagittal brain tissue sections are color-coded hindbrain–orange, midbrain–green, prefrontal cortex/isocortex–blue, basal ganglia–red, and cerebellum–gray. (a) Selected lipid classes/subclasses detected by regular MALDI-MSI negative ion mode lipid species measured as [M-H]^−^ ions (i) PE, (ii) PS, (iii) PI, and (iv) SHexCer. (b) Selected lipid classes/subclasses detected by regular MALDI-2 MSI positive ion mode (i) HexCer, (ii) SM lipid species measured as [M+H]^+^ ions, and (iii) a combined nonisobaric list of PC [M+H]^+^ and PC [M+Na]^+^ adducts. Each bar represents the average concentration from *n* = 3 biological replicates, and error bars represent ±1 standard deviation. Individual data points from each replicate are provided for each species.

Next, we deployed our multiplexed Q-MSI approach to reveal lipid concentrations within histologically defined brain regions. Brain regions were defined on the H&E-stained tissue sections, which were then coregistered with MSI data (see methods). Figure 3a shows the concentrations (pmol/mm^2^) in negative ion mode for PE, PS, PI, and SHexCer as [M-H]^−^ acquired using the Orbitrap Elite. Lipid concentrations across the presented classes had concentrations for sum-composition species varying from several hundred pmol/mm^2^ to less than 1 pmol/mm^2^. These data also highlight the effect of class-specific ionization biases. For example, the base peak in negative-ion mode is typically either PI 38:4 or SHexCer 42:2;O2 depending on the brain region; however, several PE and PS species have absolute concentrations several fold higher than both PI 38:4 or SHexCer 42:2;O2. These results are also consistent with those reported by Fitzner et al., which also showed higher amounts of PE and PS in brain tissue compared to PI and SHexCer.^55^

Figures 3b shows the Q-MSI obtained for all detected lipid species in positive ion mode using the Orbitrap Elite across SM and HexCer as [M+H]^+^ ions, and PC as either [M+H]^+^ or [M+Na]^+^ ions. For each PC species, individual adducts were chosen to avoid isobaric interference between sodiated and protonated PC lipids. Generally, this meant that PC species with few double bonds were reported as the protonated species and more unsaturated species were analyzed as the sodiated species (Supporting Information Table S3). This approach was used given that the [M+K]^+^ adduct of the PC IS could not be resolved from the [M+1] isotope of protonated PS 36:1. As expected, PC species yielded the highest concentrations with several species having concentrations >100 pmol/mm^2^. Other myelin-specific lipids such as HexCer yielded concentrations approaching 80 pmol/mm^2^ for the most abundant HexCer 42:2;O2. Region-specific concentrations of additional lipid classes and subclasses for data acquired using the Orbitrap are provided in Supporting Information Figure S11 and corresponding data using the timsTOF is provided in Supporting Information Figures S12 and S13.

To validate our quantitative technique, we compared spatial lipidomics data from this study to those of other bulk lipidomics studies that reported lipid concentrations in units comparable to our data. After accounting for section thickness and assuming a tissue density of 1 g/cm^3^, we have converted our values to match the prior literature to allow for direct comparisons, although we note that such comparisons can be difficult due to differences in the age and diet of the animals, section location, and other possible sources of variation between sample types. Eiersbrock et al.,^58^ reported concentrations of PC 34:1 of 9.07 ± 0.9 nmol/mm^3^ and 7.0 ± 1.3 nmol/mm^3^ for the WM and the molecular layer (ML), respectively. On the other hand, Choi et al.,^59^ reported concentrations of 10.06, 2.13, and 1.12 nmol/mm^3^ for PC 34:1, PC 38:4, and PC 40:6, respectively, from whole mouse brain tissue. Averaged across the entire tissue, we obtained a concentration for PC 34:1 of 16.17 nmol/mm^3^ after accounting for tissue thickness, with similar consistency also found for PC 38:4 and PC 40:6 at 2.49 and 1.06 nmol/mm^3^, respectively. Both studies also reported concentrations for PE 34:1 between 0.46 and 1.46 nmol/mm^3^ depending on the brain region, which again compares favorably with our data: 0.97 nmol/mm^3^. Eiersbrock et al., also reported a concentration of 10.2 ± 1.4 and 0.37 ± 0.181 nmol/mm^3^ for SHexCer 42:2;O2 for the WM and ML, respectively, which is consistent with the value we obtained of 1.47 nmol/mm^3^ (note that higher values are observed in the WM regions, consistent with Eiersbrock et al). Our results for PC quantitation are also generally consistent with reported values by Jadoul et al., who used a spiked tissue homogenate and an isotopically labeled PC IS to quantify PC species in thin sections of mouse brain using MALDI-MSI.^60^ Jadoul et al., reported whole section concentrations for protonated PC 34:1, PC 36:1, and PC 32:0 of 14,980 μg/g (12,282 ug/g in this work), 6,347 μg/g (3,682 ug/g in this work), and 4,594 μg/g (6,527 ug/g in this work), respectively. In addition, we have compared our absolute ratio of PC to PE lipids to data acquired using LC-MS with good agreement between the methods (Supporting Information Figure S14). Taken together, these data provide confidence that accurate concentrations reported by our multiplexed Q-MSI approach (after averaging across tissue regions for comparison) yield similar results acquired following lipid extraction quantitation using LC-MS or shotgun lipidomics, especially once considerations such as tissue water content are factored in for studies that reported lipid concentrations per wet tissue mass.

### Multiplatform Comparison on Multiplexed Q-MSI Approach

**Figure 4:**
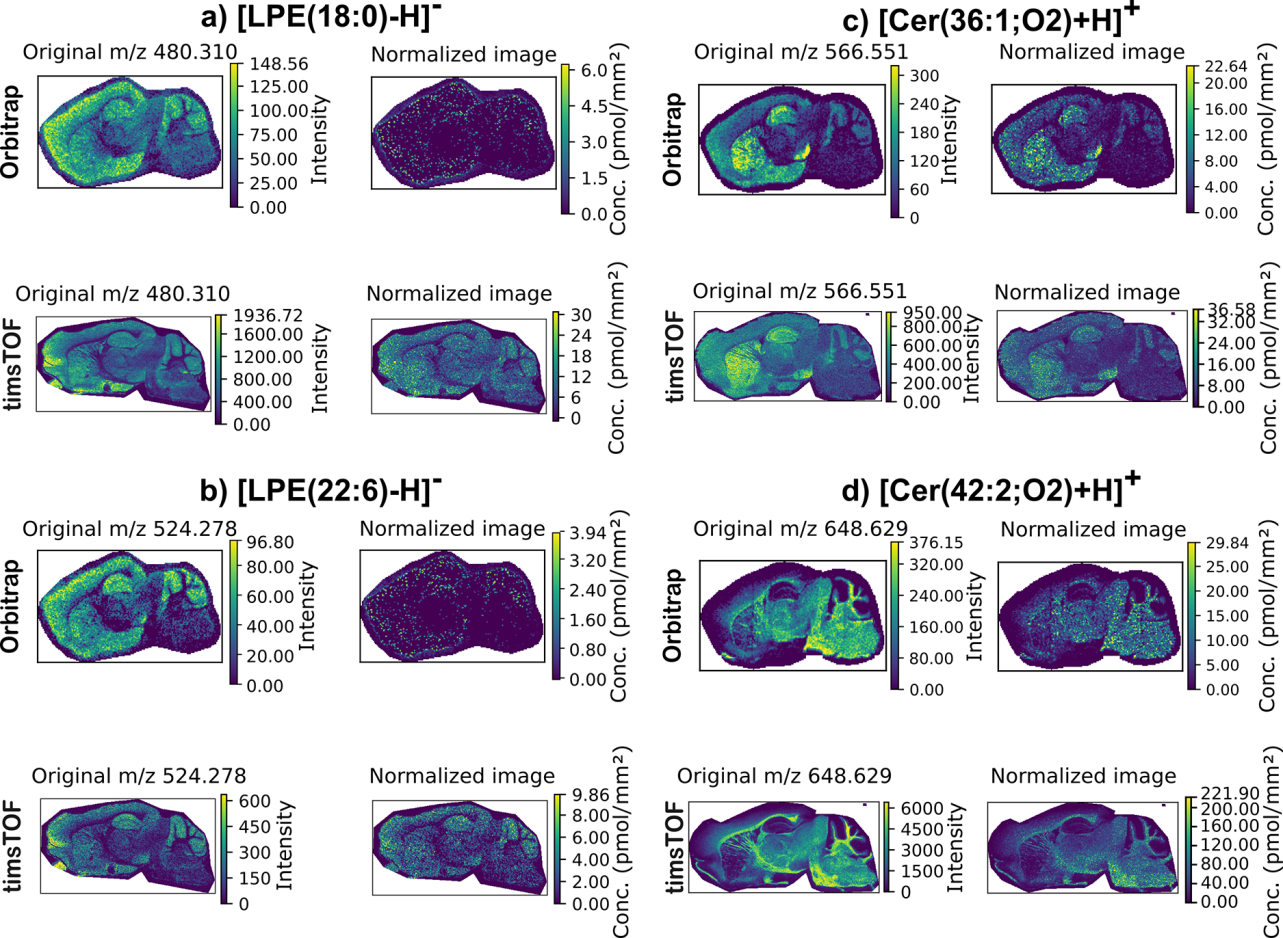
Comparison of Cer and LPE ion images obtained using the Orbitrap Elite and timsTOF systems; (a) [LPE(18:0)-H]^−^, (b)[LPE(22:6)-H]^−^, (c) [Cer(36:1;O2)+H]^+^, and (d) Cer(42:2;O2)+H]^+^. The higher quality images obtained with the timsTOF arise due to better detection of the LPE and Cer IS peaks.

Overall, similar quantitative results and ion images were observed between the Orbitrap and timsTOF data (Supporting Information Figures S10, S11, S12, and S13). The timsTOF yielded superior image quality for IS normalized ceramide and LPE species, with several examples highlighting this shown in Figure 4. This is largely due to better detection of the respective LPE and Cer IS species using the timsTOF under the employed conditions - which has an ion intensity similar to only moderately abundant endogenous LPE and Cer species and appears close to the noise limit in the Orbitrap data. The increased sensitivity for LPE and Cer could arise from an enhanced transmission of lower *m*/*z* species or may reflect an enhanced ionization resulting from a difference in ion source designs. The high concentrations reported for Cer 42:2;O2 likely arises due to in-source fragmentation of the abundant HexCer 42:2;O2, which also contributes to the ion signals. Using the HexCer IS we found the timsTOF data resulted in approximately 6% fragmentation of HexCer to Cer, compared to only ∼1.5% using the Orbitrap. This highlights the importance of considering in-source fragmentation effects when interpreting Q-MSI data and the utility of internal standard monitoring of in-source fragmentation.

**Figure 5:**
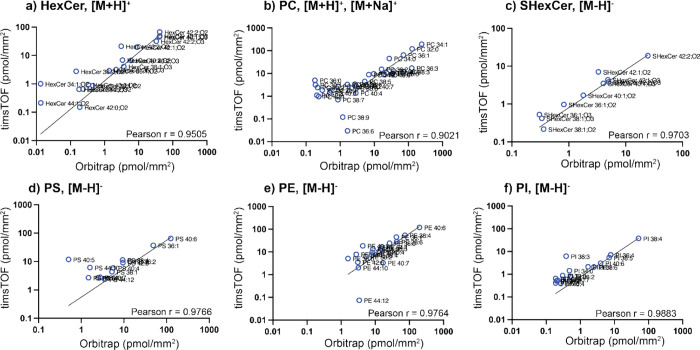
Correlation of Q-MSI data acquired using the timsTOF (mass resolution ∼50,000 @*m*/*z* 750) and Orbitrap Elite (mass resolution ∼180,000 @ *m*/*z* 750) following averaging of all on-tissue pixels for each section. Data are provided for (a) HexCer, (b) PC, (c) SHexCer, (d) PS, (e) PE, and (f) PI lipid species. Each data point is the average of *n* = 3 biological replicates measured on each system with the Pearson correlation coefficient *r* value from two-tailed tests shown as an inset. The majority of outliers can be explained by isobaric overlap encountered in the lower resolution Q-TOF data, which adds additional peak intensity in the extracted mass windows (see methods). Correlation plots for additional lipid species are provided in Supporting Information Figure S15.

We next investigated the correlation in quantitative values obtained between the Orbitrap and timsTOF instruments by comparing the concentrations across whole tissue sections. Figure 5 shows this correlation analysis for (a) SHexCer [M-H]^−^, (b) PC ([M+H/Na]^+^, (c) HexCer ([M + H]^+^, (d) PS [M-H]^−^, (e) PE [M-H]^−^, and (f) PI [M-H]^−^ lipid species. Correlation plots for additional lipid classes and subclasses can be found in Supporting Information Figure S15. These data also demonstrate the Q-MSI of lipids species covering almost 3 orders of magnitude. Encouragingly, high correlation of quantitative values (pmol/mm^2^) were obtained for most lipid classes and subclasses that were detected by both instruments. Outliers were observed, which were generally believed to be due to artificially elevated intensities in the timsTOF data given the lower mass resolution and, consequently, isobaric interferences for some lipid masses. Many of these are attributed to the common type II isobaric overlap resulting from the [M+2] isotope of lipids containing one less double bond that are not resolved on the timsTOF but are resolved on the Orbitrap (e.g., [M-H]^−^ ions of PI 38:4[^13^C_2_] and PI 38:3). Here, we have deliberately decided to show these isobaric overlaps to highlight the effect of insufficient mass resolution on Q-MSI results; however, isotope correction could be performed to correct these outliers. Other outliers can be explained by an apparent increased sensitivity for certain lipid species on one instrument platform under the employed conditions. For example, several low abundance HexCer species such as HexCer 34:1;O2 and HexCer 44:1;O2 are observed at higher concentration using the timsTOF. This can be explained by the better detection of these HexCer species using the timsTOF. On the Orbitrap system these species have single pixel intensities very close to the noise level meaning they are not detected in some pixels. This results in an underestimation of their concentration. Nonetheless, Figure 5 demonstrates that for lipids well detected by both systems without major isobaric interferences, a strong agreement in Q-MSI data is obtained using both approaches, even when the IS was deposited onto tissues in different laboratories.

## Conclusions

By developing a multiclass IS mix and building on established quantitative workflows for shotgun lipidomics, this study demonstrates an approach for Q-MSI of lipids on an omics-wide scale (i.e., performing Q-MSI for multiple lipid species across multiple classes and subclasses, simultaneously). The method can be readily adapted to other MSI modalities such as DESI, nano-DESI, IR-MALDESI, and SIMS. Using the average concentrations across tissue sections, our data are consistent with quantitative amounts per mass of tissue reported in prior bulk lipidomics studies on mouse brain, demonstrating the validity of the approach. The use of MALDI-2 also facilitated Q-MSI of lipid species not typically detected using conventional MALDI, such as glycosphingolipids.

The robustness of our workflow is demonstrated by achieving similar Q-MSI results across two different laboratories and technical operators using both a higher mass resolution but a lower throughput Orbitrap mass spectrometer and a much faster but lower mass resolution Q-TOF system. This multisite comparison yielded similar quantitative values for lipid species that are well resolved in the mass spectra using both platforms and also provided insight into the number of lipid species for which quantitative errors may be encountered if an insufficient mass resolution is available to resolve isobaric interferences. Given the sensitivity of the MALDI-2 signal to laser alignment, the use of the IS mix is also shown to correct for MALDI-2-related artifacts that can lead to striping artefacts across ion images resulting in changes in MALDI-2 ionization efficiencies across the tissue (e.g., Supporting Information Figure S4). This approach can contribute to the robustness and comparison of MALDI-2 data acquired across long studies or different laboratories. The approach can be further enhanced in the future by coupling with ion mobility methods to further remove possible isobaric interferences.

From a broader lipidomics perspective, this work provides an avenue to precisely determine lipidomes across tissue regions. With this, new metabolic information behind different pathologies, such as neurodegenerative disease or cancer, can be quantitively explored by revealing region-specific lipid compositional changes. It further creates an initial framework for the adaptation of lipid MSI to the established Lipidomics Minimal Reporting Checklist emphasizing on standardization and harmonization.^61^

## Supporting Information Available

Method for haematoxylin and eosin staining; method for LC-MRM analyses of PE and PC lipid species; Table S1: internal standard concentrations; Table S2: ions used for mass recalibration; Table S3: list of adducts used for Q-MSI of PC species; Figure S1: extracted lipid species from analysis of mouse brain tissue on an Orbitrap Elite system; Figure S2: extracted lipid species from analysis of mouse brain tissue on a timsTOF Flex system; Figure S3: Orbitrap Elite unprocessed averaged on-tissue spectra; Figure S4: histogram showing the ratio of lipid signal intensities; Figure S5: influence of IS spraying method on MSI data; Figure S6: influence of IS spraying method on acquired mass spectra; Figure S7: subtle changes in spatial localization revealed by Q-MSI; Figure S8: selected Q-MSI data in negative ion mode; Figure S9: reproducibility of quantitative mass spectrometry imaging; Figure S10: representative internal standard normalized ion images acquired using the timsTOF; Figure S11: region-specific mean lipid concentrations from Orbitrap Elite analyses; Figure S12: region-specific mean lipid concentrations from timsTOF positive mode analyses; Figure S13: region-specific mean lipid concentrations from timsTOF negative mode analyses; Figure S14: violin plot comparing PC to PE ratio obtained by MALDI-MSI vs LC-MS/MS; Figure S15: correlation of Q-MSI (PDF)
Full list of lipid species (XLSX)
