# Supplementary material for: Toward Omics-Scale Quantitative Mass Spectrometry Imaging of Lipids in Brain Tissue Using a Multiclass Internal Standard Mixture
Source: Anal Chem. 2023 Dec 11;95(51):18719–30. doi: 10.1021/acs.analchem.3c02724 (PMC11372745; doi:10.1021/acs.analchem.3c02724)
Supplement: Supplementary file 1 — ac3c02724_si_001.pdf [file ac3c02724_si_001.pdf]

Supporting Information

Towards Omics Scale Quantitative Mass Spectrometry Imaging of Lipids in Brain Tissue using a Multi-Class Internal Standard Mixture

Michiel Vandenbosch[#1], Shadrack M. Mutuku[#2], Maria José Q. Mantas[3], Nathan H. Patterson[3], Tucker Hallmark[4], Marc Claesen[3], Ron M. A Heeren[1], Nathan G. Hatcher[5], Nico Verbeeck[3], Kim Ekroos[6]* and Shane R. Ellis[2]*

[1]Maastricht MultiModal Molecular Imaging (M4I) Institute, Division of Imaging Mass Spectrometry, Maastricht University, Maastricht, 6229ER, Netherlands;

[2]Molecular Horizons and School of Chemistry and Molecular Bioscience, University of Wollongong, Wollongong, NSW 2522, Australia;

[3]Aspect Analytics NV, Genk, 3600, Belgium;

[4]Avanti Polar Lipids, Alabama, AL 35007, USA;

[5]Merck & Co., Inc., 770 Sumneytown Pk, West Point, PA, 19486, USA;

[6]Lipidomics Consulting Ltd., Esbo, 02230, Finland

# M. V and S. M. M contributed equally to this work

To whom correspondence should be addressed:

sellis@uow.edu.au

kim@lipidomicsconsulting.com

Supporting Information Content

Method for haematoxylin and eosin staining 3
Method for LC-MRM analyses of PE and PC lipid species 4
Table S1. Internal standard concentrations. 5
Table S2. Ions used for mass recalibration 6
Table S3. List of adducts used for Q-MSI of PC species 7
Figure S1. Extracted lipid species from analysis of mouse brain tissue on an Orbitrap Elite system. 8
Figure S2. Extracted lipid species from analysis of mouse brain tissue on a timsTOF Flex system. 9
Figure S3. Orbitrap Elite unprocessed averaged on-tissue spectra. 10
Figure S4. Histogram showing the ratio of lipid signal intensities. 11
Figure S5. Influence of IS spraying method on MSI data. 12
Figure S6. Influence of IS spraying method on acquired mass spectra. 13
Figure S7. Subtle changes in spatial localisation revealed by Q-MSI. 13
Figure S8. Selected Q-MSI data in negative ion mode. 14
Figure S9. Reproducibility of quantitative mass spectrometry imaging. 15
Figure S10. Representative internal standard normalized ion images acquired using the timsTOF 16
Figure S11. Region-specific mean lipid concentrations from Orbitrap Elite analyses 17
Figure S12. Region-specific mean lipid concentrations from timsTOF positive mode analyses. 18
Figure S13. Region-specific mean lipid concentrations from timsTOF negative mode analyses 19
Figure S14. Violin plot comparing PC to PE ratio obtained by MALDI-MSI vs LC-MS/MS 19
Figure S15. Correlation of Q-MSI 20

Method for haematoxylin and eosin staining

At UOW, post-MALDI imaged tissue sections were stained for histological and anatomical features by the haematoxylin and eosin (H&E) staining method. MALDI matrix was removed by immersion of slides in 100% methanol for 30 sec. Tissues were rehydrated by a series of graded ethanol washes of 95% ethanol (aq.) and 70% ethanol (aq.) and deionized water for 2 min each. Slides were stained by haematoxylin for 3 min, blued by rinsing in running tap water until clear and a 1 min wash in distilled water. Tissues were then stained in eosin for 30 sec, washed in two changes of 95% ethanol and once in 100% ethanol for 1 min each and transferred to xylene. Glass coverslip were placed onto samples by Quick hardening mounting medium. Digital optical scans of H&E-stained section were acquired at 10x magnification on a Falcon SP8 Confocal microscope (Leica Systems, Germany).

At UM, H&E staining was performed on the same 12-µm sections used for MALDI-MSI experiments. The residual matrix was removed by submerging the slides in 70% ethanol for 3 minutes. After a brief wash in Mill-Q water, the slide was re-submerged in 70% ethanol for 3 minutes, followed by Milli-Q water for 3 minutes. The slides were stained in hematoxylin for 3 minutes, followed by rinsing under running tap water for 3 minutes, then placed in eosin for 10 seconds. After placing the slides under running tap water for 1 minute, they were left in 100% ethanol for 1 minute followed by xylene for 30 seconds. The stained sections were mounted with Entellan and covered with a glass coverslip, then dried at room temperature. The stained sections were scanned with a digital scanner (Aperio CS2) at 20x magnification.

For both UM and UOW, high-resolution digital optical H&E images of tissue sections were uploaded onto a custom annotation portal for the annotation of key brain features namely prefrontal cortex, midbrain, hindbrain, basal ganglia and cerebellum. These features were co-

registered to MSI data (.imzML) for extraction of regions of interest (ROI) for downstream analyses of lipid profile distributions.

Method for LC-MRM analyses of PE and PC lipid species

Orthogonal analyses of PE and PC species in crude brain extracts was performed in a separate cohort of wild type (WT) C57BL/6N mice aged to 24 weeks at Merck & Co., Inc following in-life conditions described above for housing conditions and isolation of brain specimens. Immediately prior to LC-MS/MS analyses, fresh frozen hemi brain sections were thawed on ice and homogenized in MeOH:H_2O (1:1, v/v) buffer with volumes normalized across samples by wet tissue weight to a final value of 0.1 g/mL. Automated addition of stable isotopically labeled standards (PC 15:0_18:1d7 and PE 15:0_18:1d7, final concentrations of 10 μg/mL; Avanti Polar Lipids, Alabaster, AL) and lipid extraction was performed in 96 well plate format using a Microlab Nimbus workstation (Hamilton, Reno, Nevada) following methods described in Zhang et al, J. Lipid. Res, 2022. Briefly, lipid extraction of 100 uL of brain homogenate was performed using chloroform/MeOH + 0.1% butylated hydroxytoluene at 2:1. The plate was mixed vigorously for 30 min, and phase separation was performed by centrifugation (Sorvall Legend centrifuge, Kendro Laboratory, Germany) at 2500 rpm for 10 min at 15°C. The lower phase was transferred to a separate plate and dried under nitrogen gas at room temperature. To the remaining upper aqueous phase, 360 μl of chloroform was added and repeated as above. After centrifugation, the lower organic phase was pooled with the previous organic fraction. The final sample extracts were dried under nitrogen and reconstituted with 150 μl 2-propanol: dichloromethane (1:1, v/v) prior to analyses.

Targeted quantification of endogenous PE and PC species by LC-MRM analyses was performed as described previously by Zhang and colleagues. Briefly, analyses utilized a duo

channel UPLC system (Thermo Scientific Waltham, MA) coupled to a Sciex (Framingham, MA) 6500 triple quadrupole using electrospray ionization operating in negative mode.[1] Separation was achieved using a HALO hydrophilic interaction chromatography (HILIC) (90Å, 2.7 µm, 4.6 mm X 150 mm, Advanced Materials Technology) with mobile phases A: water containing 15 mM ammonium acetate, B: 98.5/1.5 ACN/water containing 15 mM ammonium acetate. Targeted peak area integration was performed manually using MultiQuant (Version 3.03, Sciex).

Table S1. Internal standard concentrations. Spraying conditions: Flow rate = 0.06 mL/min, time = 1.55 min, surface area sprayed = 1,150 mm^2, number of layers = 16 passes, dilution factor = 0.10.

Name	Molecular Formula	Monoisotopic Mass (Da)	Average Mass (Da)	Concentration (µg/mL)	Total (µg/mm^2)	Picomoles/ mm^2
15:0-18:1 (d7) PA (Na salt)	C36H61D7O8PNa	689.4989	689.927	111.00	0.144	20.82
15:0-18:1 (d7) PE	C38H67D7NO8P	710.5591	711.013	100.00	0.129	18.20
15:0-18:1 (d7) PG (Na salt)	C39H67D7O10PNa	763.5357	764.006	49.00	0.063	8.30
15:0-18:1 (d7) PI (NH4 salt)	C42H75D7O13P	832.5932	847.116	23.00	0.030	3.51
17:0-16:1 (d5) PS (Na salt)	C39H68D5NO10PNa	774.5184	775.000	105.00	0.136	17.53
C12 Mono-Sulfo Galactosyl(β) Ceramide (d18:1/12:0)	C36H69N1O11S	723.4591	741.029	19.00	0.025	3.32
17:0 (d5) Lyso PE	C22H41D5NO7P	472.3326	472.610	3.00	0.004	0.82
15:0-18:1 (d7) PC	C41H73D7NO8P	752.6061	753.093	161.00	0.208	27.66
17:0 (d5) Lyso PC	C25H47D5NO7P	514.3795	514.700	3.00	0.004	0.75
18:1-18:1 SM (d9)	C41H72D9N2O6P	737.6397	738.140	31.00	0.040	5.43
C15 Lactosyl(β) Ceramide (d18:1-d7/15:0)	C45H78D7NO13	854.6460	855.210	13.00	0.017	1.97
C18 Ceramide-d7 (d18:1/18:0)	C36H64D7NO3	572.5873	572.997	11.00	0.014	2.48
C17 Glucosyl(β) Ceramide (d18:1/17:0)	C41H79NO8	713.5806	714.068	133.00	0.172	24.10

Table S2. Ions used for mass recalibration

EN- Endogenous Lipid, IS- Internal Standard

Positive Ion Mode					
Lipid	*Type*	*Molecular Formula*	*Ion Adduct*	*m/z*	*Instrument*
PE 15:0-18:1(d7)	IS	C38H67D7NO8P	$[M+H]^+$	711.56642	Elite
GluCer d18:1/17:0	IS	C41H79NO8	$[M+H]^+$	714.58784	Elite , timsTOF
PC 15:0-18:1(d7)	IS	C41H73D7NO8P	$[M+H]^+$	753.61337	Elite
PE 40:6	EN	C45H78NO8P	$[M+H]^+$	792.55378	Elite
**Negative Ion Mode**					
Lipid	*Type*	*Molecular Formula*	*Ion Adduct*	*m/z*	*Instrument*
SHexCer d18:1/12:0 (NH_4 salt)	IS	C36H72N2O11S	$[M-NH_4]^-$	722.45186	Elite , timsTOF
PI 38:4	EN	C47H83O13P	$[M-H]^-$	885.54985	Elite
PI 15:0-18:1(d7) (NH_4 salt)	IS	C42H75D7NO13P	$[M-NH_4]^-$	828.56249	Elite
SHexCer d42:2	EN	C48H91NO11S	$[M-H]^-$	888.62401	Elite

Table S3. List of adducts used for Q-MSI of PC species. Adducts chosen to reduce isobaric interferences. The corresponding normalization IS mass is shown for each adduct type. ppm – parts per million.

Theoretical	Measured	Formula	Species Level ID	Adduct	Normalization *m/z*	ppm Error
706.5381	706.5381	C38H76NO8P	PC 30:0	$[M+H]^+$	753.6134	0.00
730.5381	730.5381	C40H76NO8P	PC 32:2	$[M+H]^+$	753.6134	0.00
732.5538	732.5538	C40H78NO8P	PC 32:1	$[M+H]^+$	753.6134	0.00
734.5694	734.5694	C40H80NO8P	PC 32:0	$[M+H]^+$	753.6134	0.00
758.5694	758.5695	C42H80NO8P	PC 34:2	$[M+H]^+$	753.6134	0.13
760.5851	760.5851	C42H82NO8P	PC 34:1	$[M+H]^+$	753.6134	0.00
762.6007	762.6004	C42H84NO8P	PC 34:0	$[M+H]^+$	753.6134	-0.39
786.6007	786.6007	C44H84NO8P	PC 36:2	$[M+H]^+$	753.6134	0.00
788.6164	788.6163	C44H86NO8P	PC 36:1	$[M+H]^+$	753.6134	-0.13
790.6320	790.6322	C44H88NO8P	PC 36:0	$[M+H]^+$	753.6134	0.25
800.5201	800.5202	C44H76NO8P	PC 36:6	$[M+Na]^+$	775.5953	0.12
804.5514	804.5513	C44H80NO8P	PC 36:4	$[M+Na]^+$	775.5953	-0.12
806.5670	806.5689	C44H82NO8P	PC 36:3	$[M+Na]^+$	775.5953	2.36
814.6321	814.6311	C46H88NO8P	PC 38:2	$[M+H]^+$	753.6134	-1.23
816.6477	816.6474	C46H90NO8P	PC 38:1	$[M+H]^+$	753.6134	-0.37
822.5044	822.5044	C46H74NO8P	PC 38:9	$[M+Na]^+$	775.5953	0.00
824.5201	824.5197	C46H76NO8P	PC 38:8	$[M+Na]^+$	775.5953	-0.49
826.5357	826.5357	C46H78NO8P	PC 38:7	$[M+Na]^+$	775.5953	0.00
828.5514	828.5512	C46H80NO8P	PC 38:6	$[M+Na]^+$	775.5953	-0.24
830.5670	830.5672	C46H82NO8P	PC 38:5	$[M+Na]^+$	775.5953	0.24
832.5827	832.5830	C46H84NO8P	PC 38:4	$[M+Na]^+$	775.5953	0.36
834.6007	834.6002	C46H86NO8P	PC 38:3	$[M+Na]^+$	775.5953	-0.60
842.6633	842.6626	C48H92NO8P	PC 40:2	$[M+H]^+$	753.6134	-0.83
844.6790	844.6782	C48H94NO8P	PC 40:1	$[M+H]^+$	753.6134	-0.95
852.5514	852.5497	C48H80NO8P	PC 40:8	$[M+Na]^+$	775.5953	-1.99
854.5670	854.5668	C48H82NO8P	PC 40:7	$[M+Na]^+$	775.5953	-0.23
856.5827	856.5824	C48H84NO8P	PC 40:6	$[M+Na]^+$	775.5953	-0.35
858.5983	858.5985	C48H86NO8P	PC 40:5	$[M+Na]^+$	775.5953	0.23
860.6140	860.6141	C48H88NO8P	PC 40:4	$[M+Na]^+$	775.5953	0.12
870.6946	870.6943	C50H96NO8P	PC 42:2	$[M+H]^+$	753.6134	-0.34
872.7103	872.7101	C50H98NO8P	PC 42:1	$[M+H]^+$	753.6134	-0.23

Supporting Figure(s).

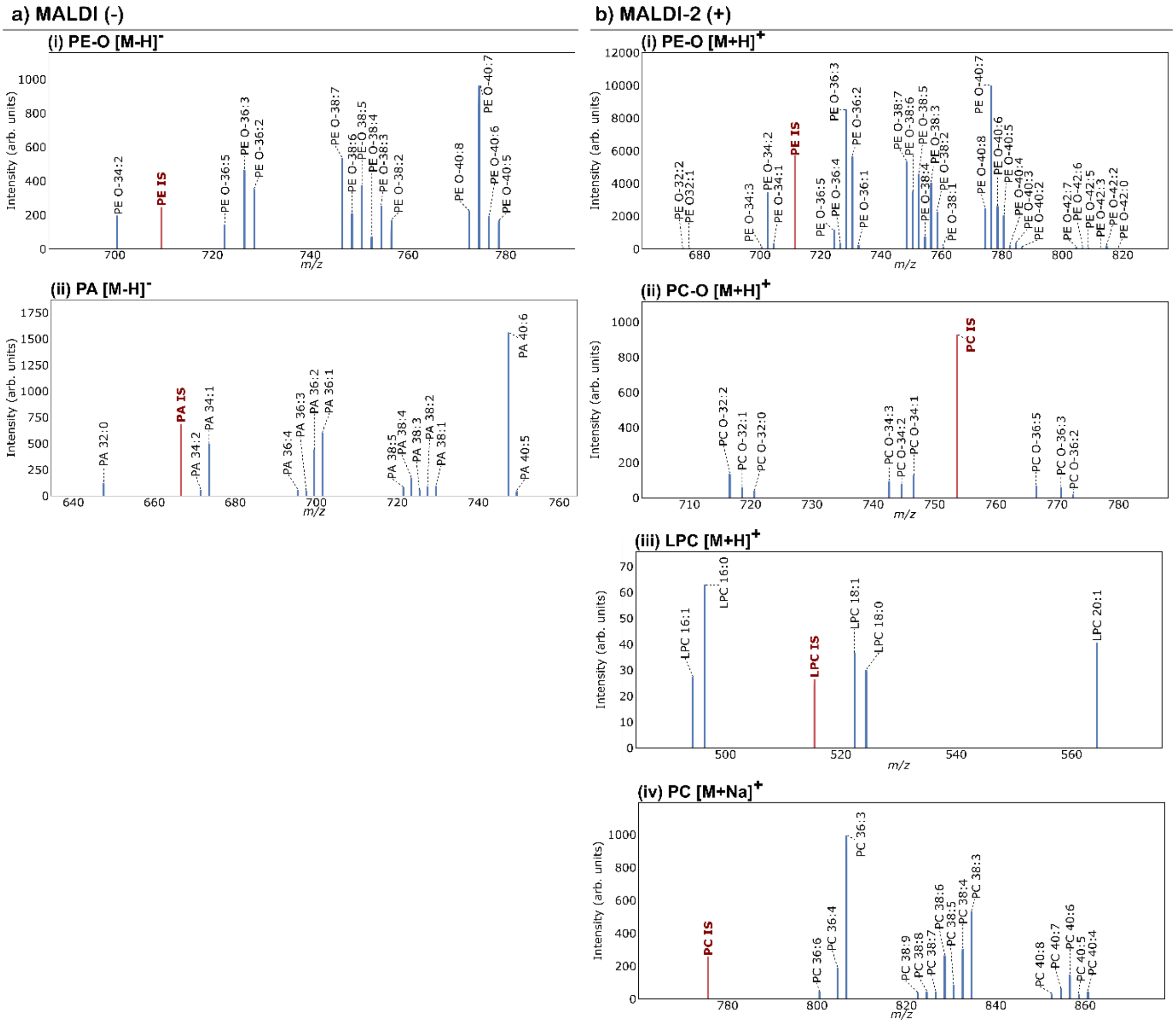

Figure S1. Extracted lipid species from analysis of mouse brain tissue on an Orbitrap Elite system. Reference IS peak shown in red and endogenous lipid species shown in blue; (a) MALDI-MSI in negative mode: $[M-H]^-$ ions for (i) PE-O and (ii) PA. (b) Laser post ionization MALDI-2 MSI in positive mode: $[M+H]^+$ ions for (i) PE-O, (ii) PC-O, (iii) LPC and $[M+Na]^+$ ions for PC.

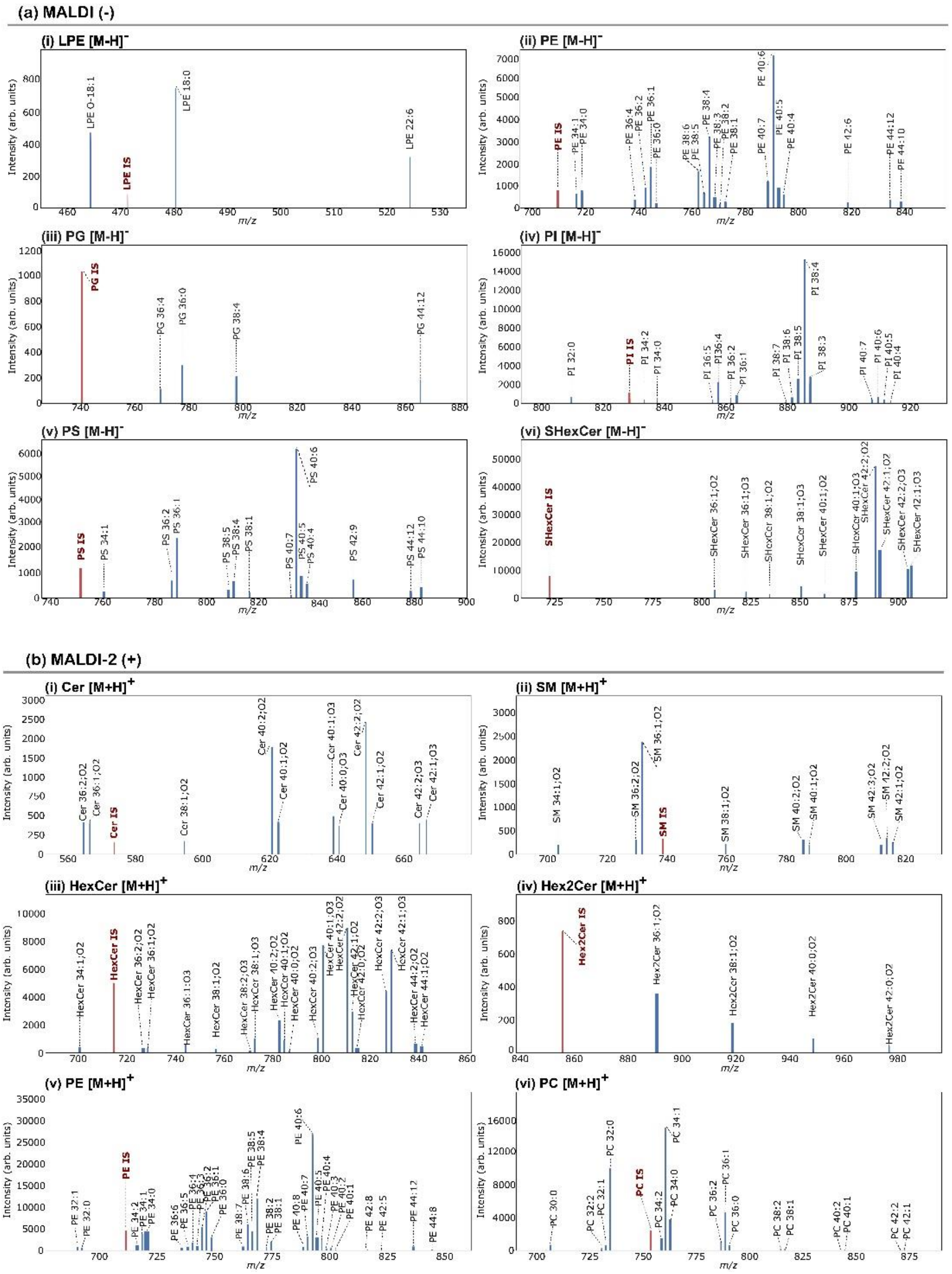

Figure S2. Extracted lipid species from analysis of mouse brain tissue on a timsTOF Flex system. Reference internal standard (IS) peak shown in red and endogenous lipid species shown in blue; (a) Regular MALDI-MSI in negative ion mode [M-H]$^-$ ions (i) LPE, (ii) PE (iii) PG, (iv) PI, (v) PS and (vi) SHexCer . (b) Laser post ionization in positive mode: [M+H]$^+$ ions for (i) Cer, (ii) SM (iii) HexCer, (iv) Hex2Cer, (v) PE and (vi) PC.

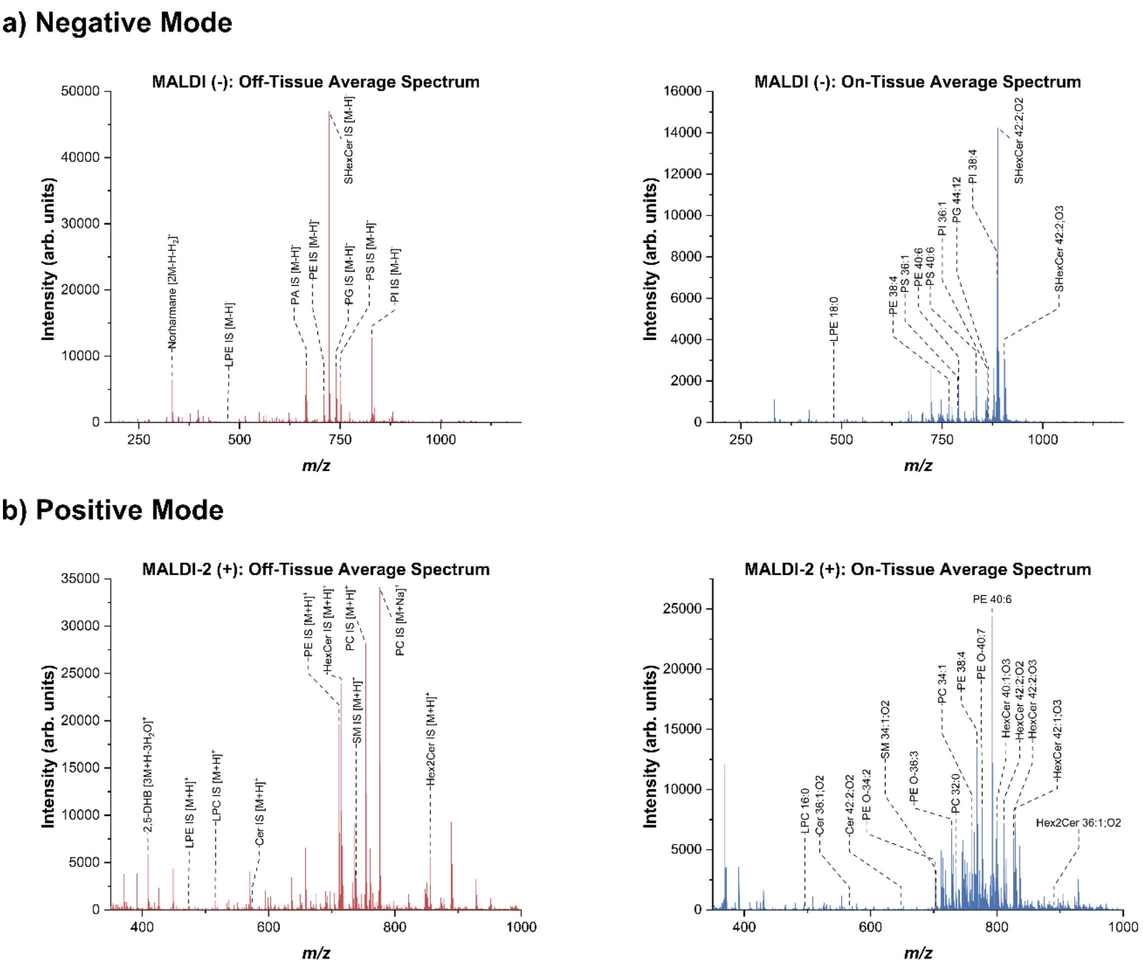

Figure S3. Orbitrap Elite unprocessed averaged on-tissue spectra. (a) Negative mode: Left panel; off-tissue showing norharmane matrix ion peak and internal standard peaks for LPE, PA, PE, SHexCer, PG, PS and PI sub-classes detected as $[M-H]^-$ ions; Right panel, on-tissue averaged spectra from a whole brain sagittal section with a variety of endogenous lipid species labelled. (b) Positive mode: Left panel, off-tissue showing DHB matrix ion peak and internal standard peaks for LPE, LPC, PE, HexCer, SM, PC and Hex2Cer as $[M+H]^+$ ions with PC also shown as $[M+Na]^+$ ion; Right panel on-tissue averaged spectra from a whole brain sagittal section with a variety of endogenous lipid species labelled.

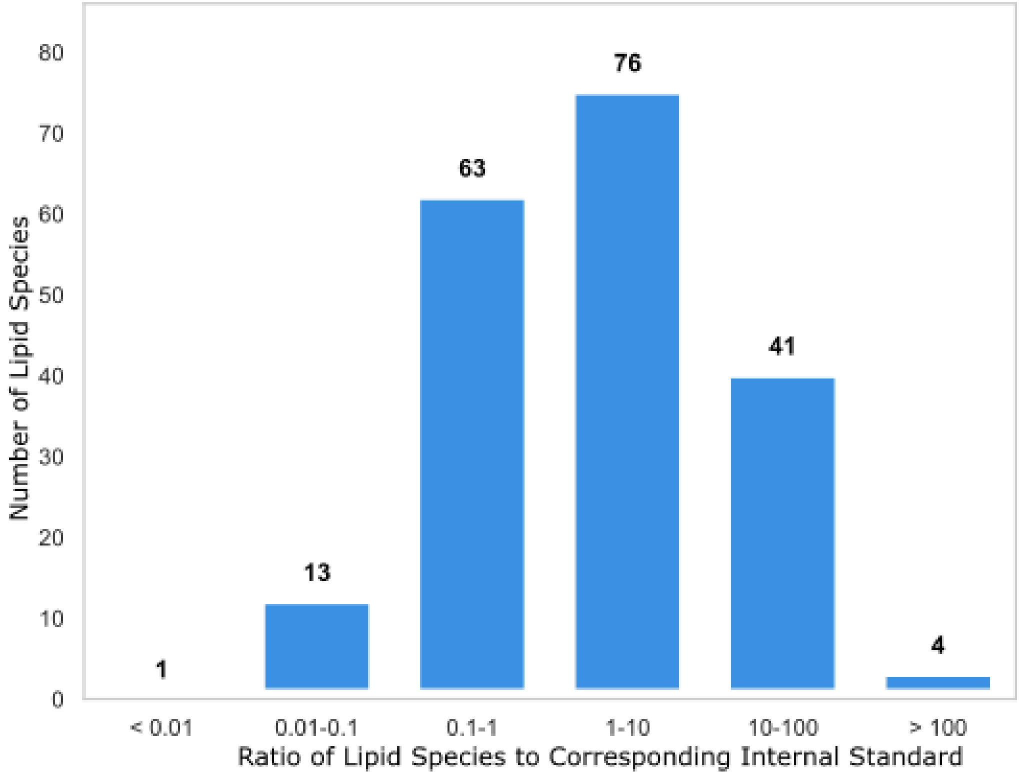

Figure S4. Histogram showing the ratio of lipid signal intensities to their corresponding internal standard in the averaged on-tissue spectrum from the Orbitrap Elite data. Note the phosphatidylethanolamine (PE) species; PE, PE-O, LPE and LPE-O species correspond to those detected as $[M\text{-}H]^-$ ions.

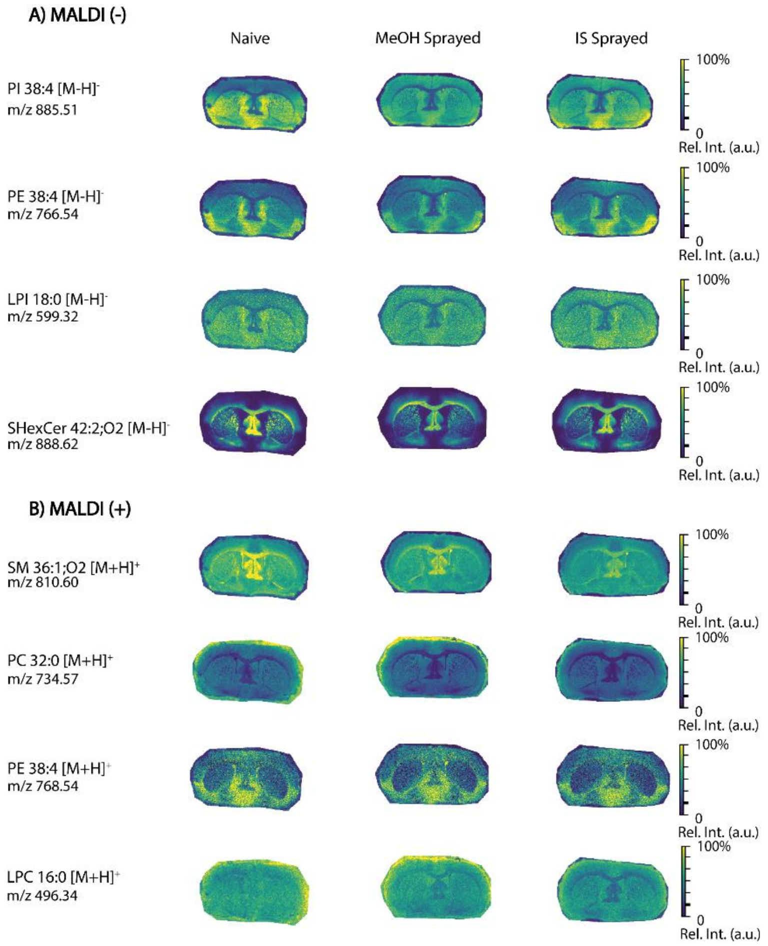

Figure S5. Influence of IS spraying method on MSI data. Different lipids species detected in (a) negative ion mode and (b) positive ion mode using regular MALDI. For each lipid species the sample prepared by spraying only matrix is on the left, the ion image after spraying with methanol followed by matrix is shown in the centre and the ion image sprayed with IS mixture follows by matrix shown on the right. Intensities for each lipid species were selected using an *m/z* window of ±12.0 ppm compared to the theoretical *m/z* of the lipid species. (a) Upper panel timsTOF MALDI-MSI negative ion mode detected as $[M\text{-}H]^-$; PI 38:4, PE 38:4, LPI 18:0 and SHexCer 42:2;O2. (b) Lower panel timsTOF MALDI MSI positive ion mode detected as $[M+H]^+$, PC 32:0. SM 36:1;O2,, PE 38:4, and LPC 16:0. All images are TIC normalised.

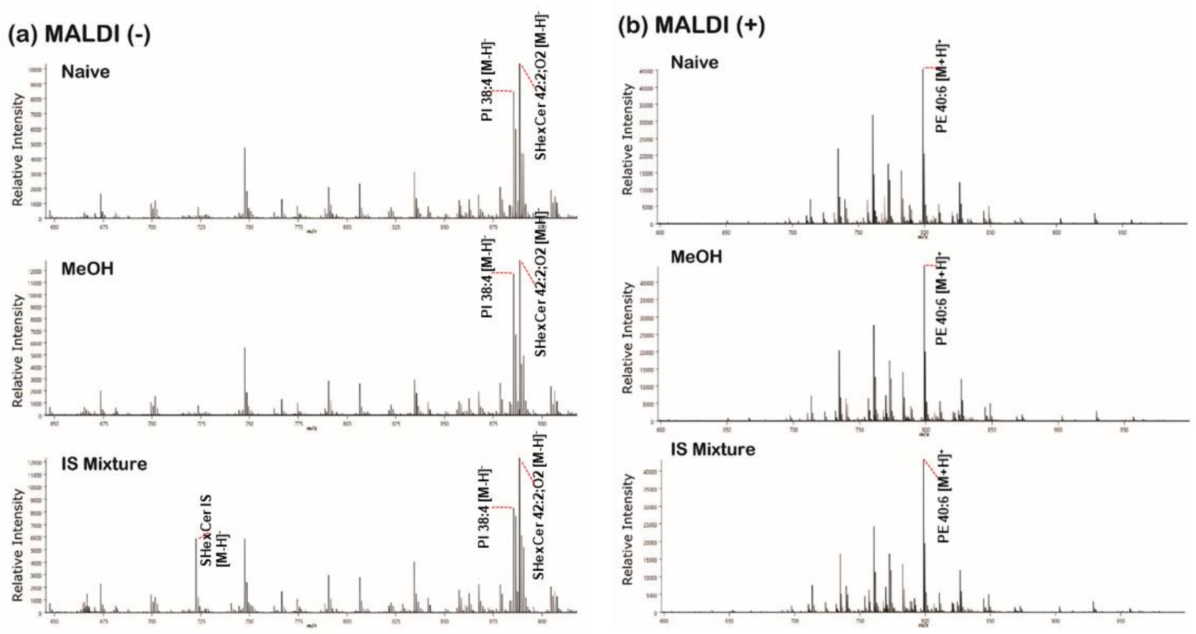

Figure S6. Influence of IS spraying method on acquired mass spectra. Averaged mass spectra acquired using the acquired using the timsTOF in (a) negative ion mode and (b) positive ion mode following (top) matrix application, (middle) spraying with methanol followed by matrix application and (bottom) spraying with IS mix followed by matrix.

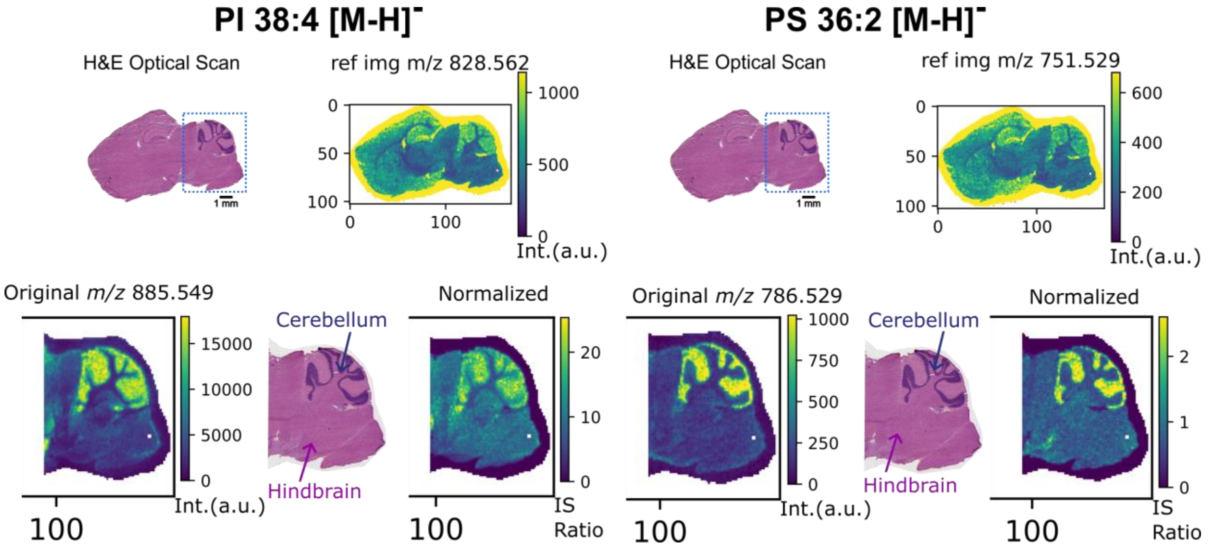

Figure S7. Subtle changes in spatial localisation revealed by Q-MSI. Top panel; Left, [PI (38:4)-H]$^-$ *m/z* 885.5499 original ion image compared to IS normalized image reference *m/z* 828.5625. Right, [PS (36:42-H]$^-$ *m/z* 786.5291 original ion image compared to IS normalized image reference *m/z* 751.5291. Bottom panel; Blue outline region zoom into brain stem and cerebellum area depicting subtle increase in [PI(38:4)-H]$^-$ *m/z* 885.5499 and [PS (36:4)-H]$^-$ *m/z* 786.5291 within the hindbrain after IS normalization.

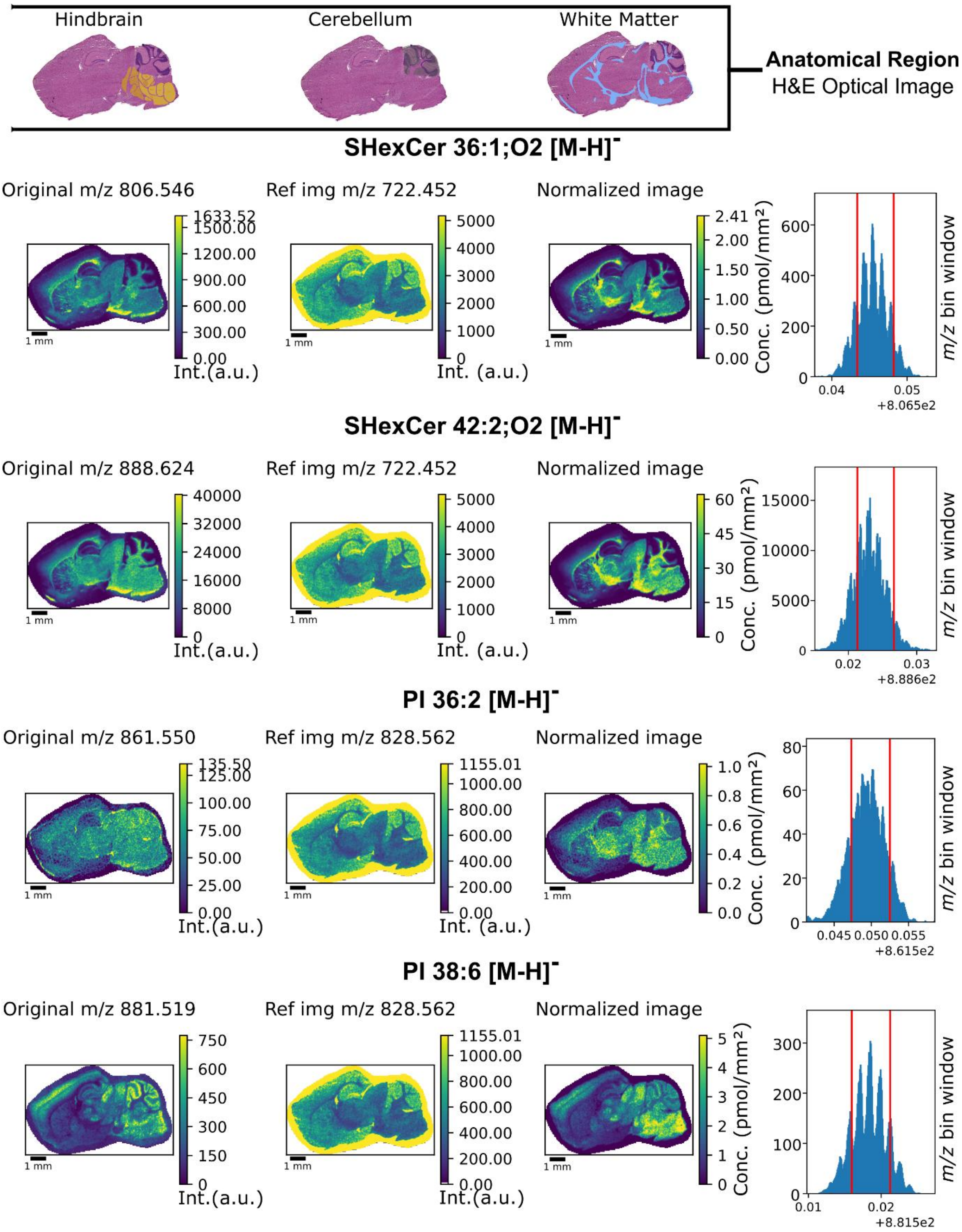

Figure S8. Selected Q-MSI data in negative ion mode. MALDI-MSI of PI 36:2, PI 38:6, and SHexCer 36:1;O2 and SHexCer 38:1;O2 measured in negative ion mode. The right hand panel shows the 3 ppm selection window used for each species.

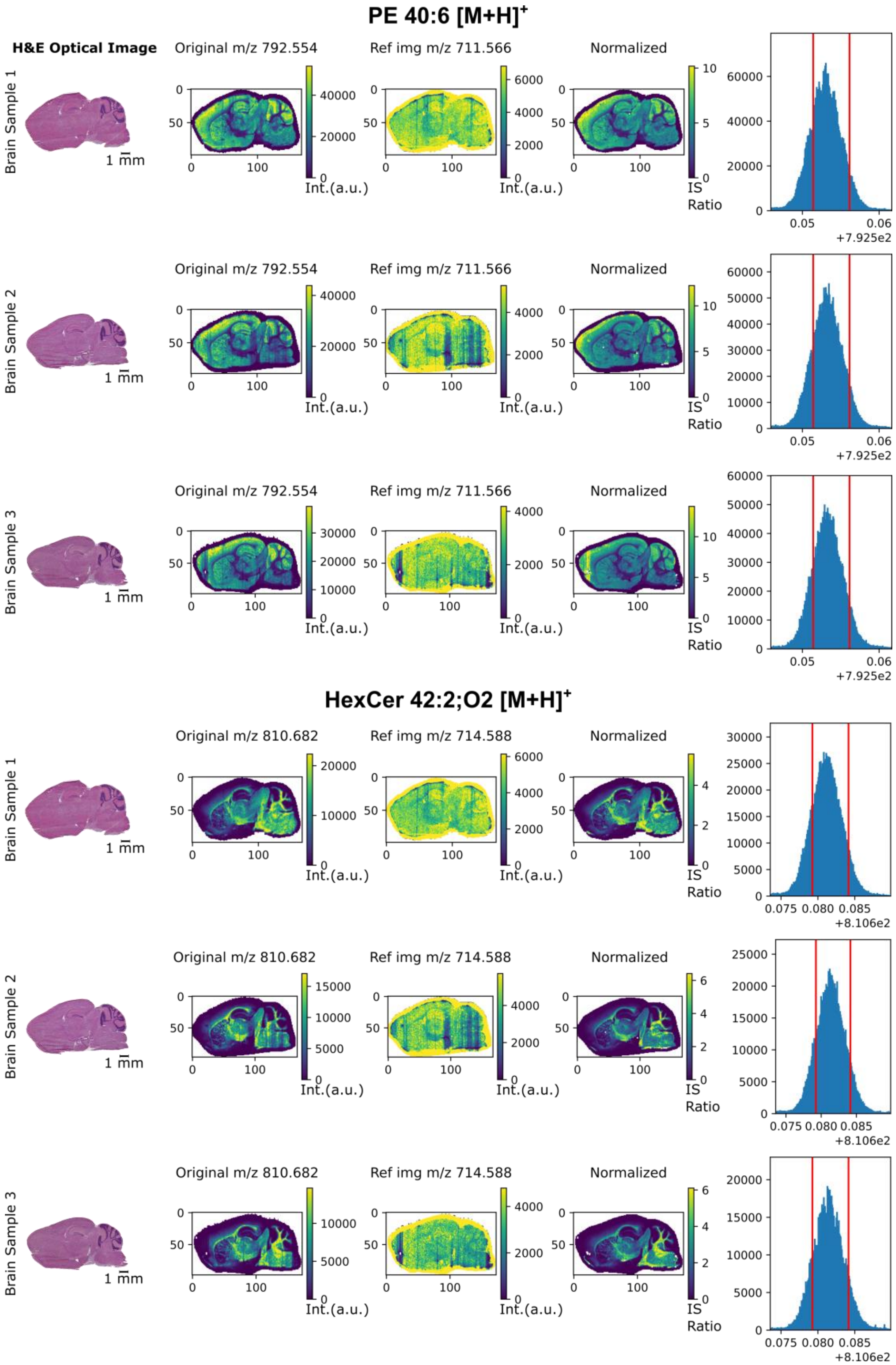

Figure S9. Reproducibility of quantitative mass spectrometry imaging. Figure shows PE 40:6 and myelin-rich HexCer 42:2;O2 species measured in positive-ion mode using MALDI-2. Data is generated from 3 biological replicates. IS normalized m/z images have significantly less stripes or line streaks in comparison to the original *m/z* images due to the correction of MALDI-2 artefacts (refer to main text). The right hand panel shows the 3 ppm selection window used for each species.

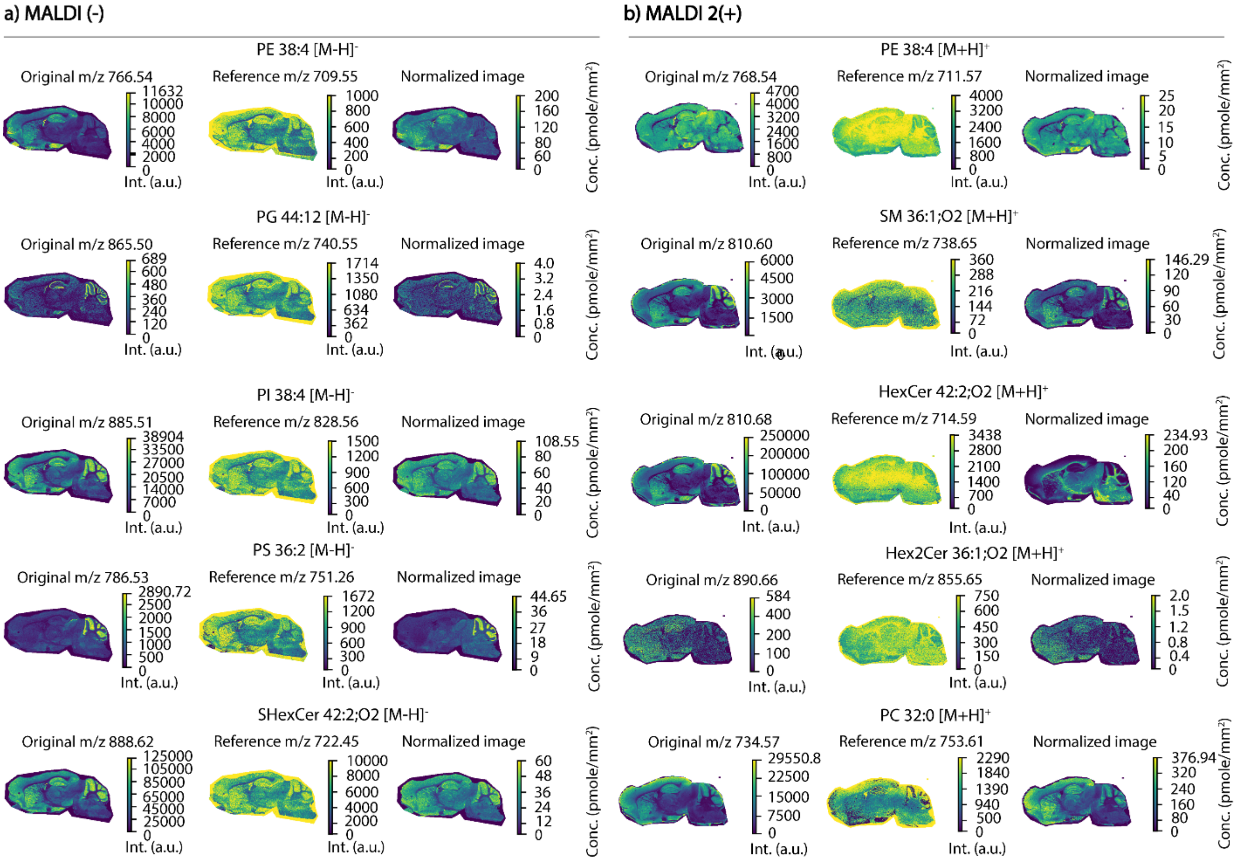

Figure S10. Representative internal standard normalized ion images acquired using the timsTOF. Figure shows different lipids species detected in (a) negative ion mode using MALDI and (b) positive ion mode using MALDI-2. For each lipid species the original ion image is shown on the left, the class-specific internal standard ion image is shown in the centre and the IS normalized ion image is shown on the right. Intensities for each lipid species were selected using an *m/z* window of ±12.0 ppm compared to the theoretical *m/z* of the lipid species (a) Left panel timsTOF MALDI-MSI negative ion mode detected as $[M-H]^-$; PE 38:4, PG 44:12, PI 38:4, PS 36:2 and SHexCer 42:2;O2. (b) Right panel timsTOF MALDI-2 MSI positive ion mode detected as $[M+H]^+$, PE 38:4, SM 36:1;O2, HexCer 42:2;O2, HexCer 42:2;O2 and PC 32:0.

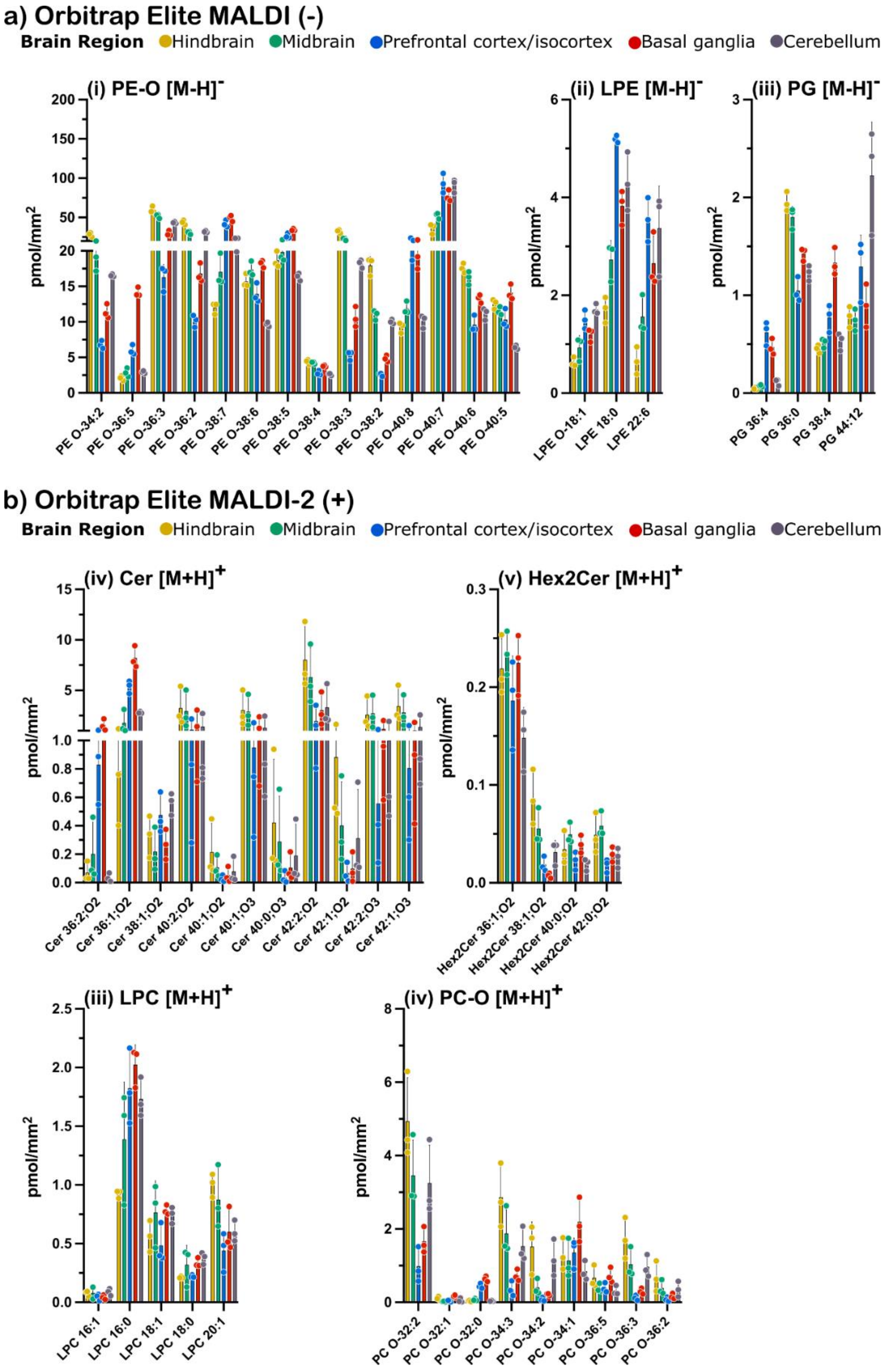

Figure S11. Region-specific mean lipid concentrations from Orbitrap Elite analyses. Regions of interest (ROI) from sagittal brain tissue sections are colour-coded hindbrain – orange, midbrain – green, prefrontal cortex/isocortex – blue, basal ganglia – red, and cerebellum – grey. (a) Quantitative MALDI-MSI negative mode $[M-H]^-$ ions for (i) PE-O, (ii) LPE/LPE-O and (iii) PG sub-classes of lipids. (b) Quantitative MALDI-2 MSI positive mode $[M+H]^+$ ions for (i) Cer, (ii) Hex2Cer, (iii) LPC and (iv) PC-O species.

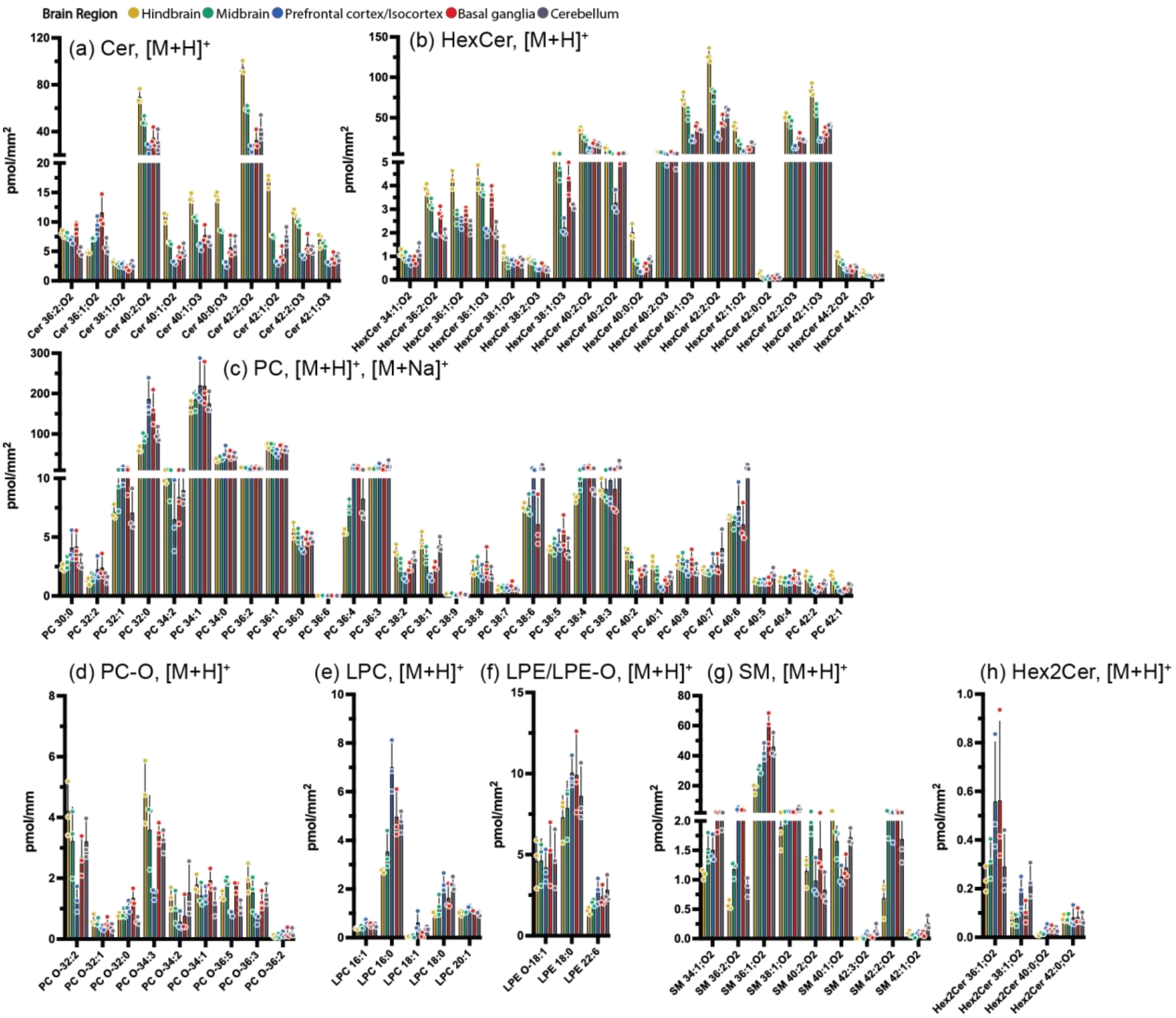

Figure S12. Region-specific mean lipid concentrations from timsTOF positive mode analyses. Regions of interest (ROI) from sagittal brain tissue sections are colour-coded hindbrain – orange, midbrain – green, prefrontal cortex/isocortex – blue, basal ganglia – red, and cerebellum – grey. Quantitative MALDI-2 MSI positive mode $[M+H]^+$ ions for (a) Cer, (b) HexCer, (c) PC, (d) PC-O, (e) SM, (f) LPC, (g) LPE/LPE-O species and (h) Hex2Cer species.

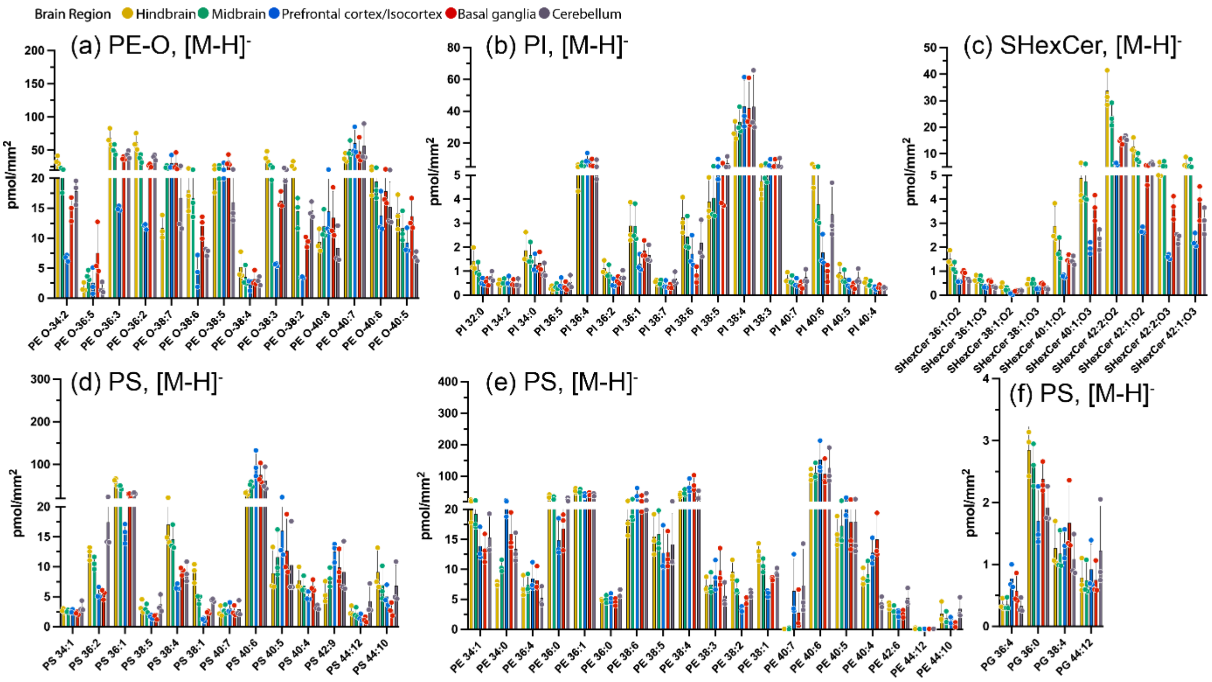

Figure S13. Region-specific mean lipid concentrations from timsTOF negative mode analyses. Regions of interest (ROI) from sagittal brain tissue sections are colour-coded hindbrain – orange, midbrain – green, prefrontal cortex/isocortex – blue, basal ganglia – red, and cerebellum – grey. Quantitative MALDI-MSI negative mode [M-H]- ions for (a) PE, (b) PG, (c) PE-O, (d), PI, (e) PS and (f) SHexCer classes/sub-classes of lipids.

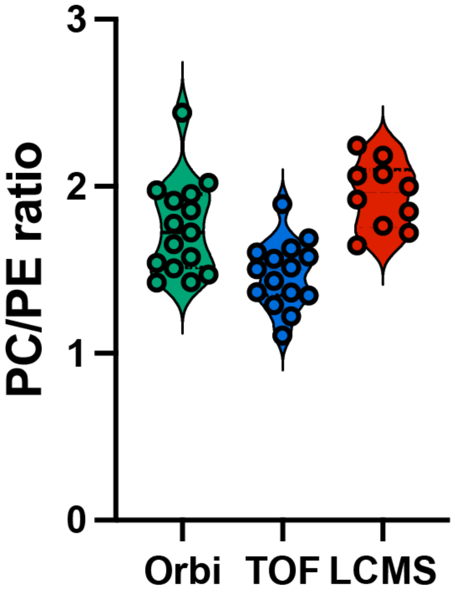

Figure S14. Violin plot comparing PC to PE ratio obtained by MALDI-MSI vs LC-MS/MS**.** Total PC and PE were obtained by summing up all class-specific species from respective analyses. PC to PE ratio was determined by dividing the total class levels. For MALDI-MSI, individual points represent a single measurement from a particular region, and for LC-MS/MS, measurements from extracted brain-homogenates of ten individual wild-type mice aged 24 weeks.

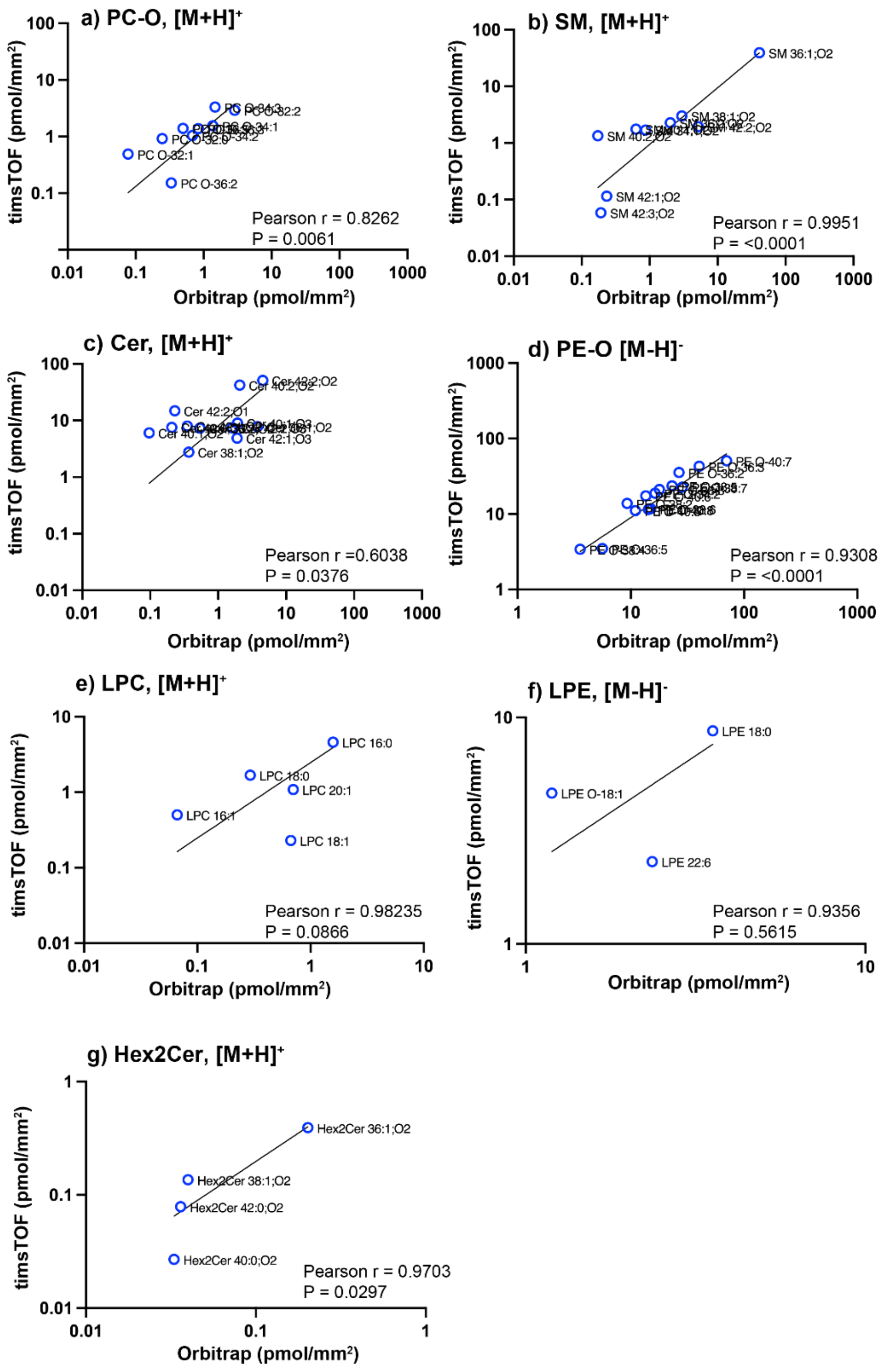

Figure S15. Correlation of Q-MSI data acquired using the Orbitrap and timsTOF following averaging of all on-tissue pixels for each section. Data is provided for (a) PC-O, [M+H]$^+$, (b) SM, [M+H]$^+$ (c) Cer, [M+H]$^+$ and (d) PE-O, [M-H]$^-$, (e) LPC, [M+H]$^+$, (f) LPC, [M-H]$^-$ and (g) Hex2Cer, [M+H]$^+$. Each data point is the average of n=3 biological replicates measured on each system. The majority of outliers can be explained by isobaric overlap encountered in the lower resolution Q-TOF data which adds additional peak intensity in the extracted mass windows (see methods), or by the increased sensitivity of one system for a given lipid class, particularly for species that appear at low intensity that is close to the noise level in one or both systems.

References

1 Zhang, N. R.; Hatcher, N. G.; Ekroos, K.; Kedia, K.; Kandebo, M.; Marcus, J. N.; Smith, S. M.; Bateman, K. P.; Spellman, D. S. Validation of a multiplexed and targeted lipidomics assay for accurate quantification of lipidomes. *J. Lipid Res.* **2022**, *63* (6), 100218.
